# GRSF1 antagonizes age-associated hypercoagulability via modulation of fibrinogen mRNA stability

**DOI:** 10.1038/s41419-023-06242-9

**Published:** 2023-11-03

**Authors:** Doudou Liu, Chenzhong Xu, Ze Gong, Yijie Zhao, Zhiqiang Fang, Xiaoli Rao, Qingyu Chen, Guodong Li, Wei Kong, Jun Chen

**Affiliations:** 1 Peking University Research Center on Aging, Beijing Key Laboratory of Protein Posttranslational Modifications and Cell Function, Department of Biochemistry and Biophysics, Department of Integration of Chinese and Western Medicine, School of Basic Medical Science, Peking University, 100191 Beijing, China; 2 School of Basic Medical Sciences, Shenzhen University, 518055 Shenzhen, China; 3 State Key Laboratory of Vascular Homeostasis and Remodeling, Department of Physiology and Pathophysiology, School of Basic Medical Sciences, Peking University, 100191 Beijing, China; 4 Hwamei College of Life and Health Sciences, Zhejiang Wanli University, 315100 Ningbo, China; 5 Department of Laboratory Animal Science, Peking University Health Science Center, 100191 Beijing, China

**Keywords:** Ageing, Thrombosis, RNA

## Abstract

Age-associated hypercoagulability is accompanied by the increase of plasma levels of some coagulation factors including fibrinogen which may contribute to the increased risk of cardiovascular, cerebrovascular, and thrombotic diseases in elderly people. However, the underlying mechanism of increased plasma fibrinogen concentration during aging is still elusive. GRSF1 belongs to the heterogeneous nuclear ribonucleoproteins F/H (hnRNP F/H) subfamily. Here, we report that GRSF1 attenuates hypercoagulability via negative modulation of fibrinogen expression. We demonstrated that GRSF1 negatively regulated fibrinogen expression at both mRNA and protein levels. GRSF1 directly interacted with the coding region (CDS) of FGA, FGB, and FGG mRNAs, and decreased their stability thus mitigating fibrinogen expression. We further identified that only a few G-tracts within the Fib C domain of FGA, FGB, and FGG CDS and the qRRM2 domain of GRSF1 were required for their interaction. Moreover, we confirmed hypercoagulability and the decrease of GRSF1 expression level during mice aging. Functionally, GRSF1 overexpression in old mice liver decreased fibrinogen plasma level, reduced hypercoagulability, and mitigated blood coagulation activity, whereas GRSF1 knockdown in young mice liver increased fibrinogen plasma level and promoted blood coagulation activity. Collectively, our findings unveil a novel posttranscriptional regulation of fibrinogen by GRSF1 and uncover a critical role of GRSF1 in regulating blood coagulation activity.

## Introduction

Aging is associated with a variety of hemostasis changes that overall reflect a hypercoagulable state compared with earlier age. It is well established that the plasma concentrations of some coagulation factors, including fibrinogen, factor V, VII, VIII, IX, von Willebrand factor (vWF), and so on, increase progressively with age in healthy humans [1–3]. Moreover, these hemostasis changes are generally independent of gender [4, 5]. Fibrinogen is the most abundant blood coagulation factor synthesized in the liver. Fibrinogen plasma level increases with age not only in humans but also in animal models, and its level in healthy subjects is expected to increase by 10 mg/dL per decade [4–8]. Elevated plasma fibrinogen levels may partially explain the increased risk of cardiovascular, cerebrovascular, and thrombotic diseases observed in elderly people [9–13]. However, the mechanism of increased plasma fibrinogen level and hypercoagulability during aging has not been fully elucidated.

Fibrinogen is a hexameric homodimer consisting of two sets of three different polypeptide chains designated Aα, Bβ and γ, which are encoded by three separate genes FGA, FGB, and FGG, respectively [14–16]. Fibrinogen plays important roles in both primary and secondary hemostasis [17, 18]. Fibrinogen is also a type II acute phase reactant that participates in multiple pathological processes such as cardiovascular, cerebrovascular, and thrombotic diseases, as well as Alzheimer’s disease, multiple sclerosis, etc. [19–24]. Fibrinogen gene expression is regulated by transcription factors HNF4α, HNF1, and C/EBP at the transcriptional level [25, 26], and by CTCF at the epigenetic level [27], as well as by microRNA from hsa-miR-29 and hsa-miR-409-3p family at posttranscriptional level [28]. As an acute phase protein, fibrinogen expression is also activated by interleukin-6 (IL-6)-STAT3 axis [25, 26]. However, the regulatory mechanism of fibrinogen gene expression has not been fully elucidated.

The guanine-rich RNA sequence binding factor 1 (GRSF1) is an RNA-binding protein (RBP) and belongs to the heterogeneous nuclear ribonucleoprotein (hnRNP) F/H subfamily. GRSF1 contains three quasi-RNA recognition motifs (qRRMs) that preferentially bind to G-rich sequences of target mRNAs, thereby participating in various posttranscriptional regulation of RNAs, including RNA translation, stability, splicing, and polyadenylation [29–32]. GRSF1 localizes in the nucleus, cytoplasm and mitochondria, and is involved in various biological processes such as mitochondrial RNA processing and trafficking [33], mitochondrial ribosome biosynthesis [34–36], erythropoiesis [37], redox homeostasis [38], and viral infection [39–41]. GRSF1 also regulates cellular senescence and may be involved in age-related skeletal muscle endurance decline [42–45]. However, the targets and pathophysiological roles of GRSF1 need to be further understood.

In this study, we identify GRSF1 as a negative fibrinogen regulator that can bind to fibrinogen mRNAs CDS region and reduce its stability, thus decreasing fibrinogen expression at the posttranscriptional level. Functionally, we show that GRSF1 overexpression in the liver decreases plasma fibrinogen levels and reduces hypercoagulability in old mice.

## Materials and methods

### Cell culture

HEK293T, LO2, and HepG2 cells were cultured in DMEM supplemented with 10% fetal bovine serum (FBS, Hyclone) and penicillin/streptomycin antibiotics. Cells were under standard cell culture conditions (37 °C, 5% CO_2_, humidified atmosphere) and passaged every 2–3 days by trypsinization at a confluency of 80–90%. All cell cultures were routinely checked for mycoplasma contamination and confirmed negative.

### Gene chip microarray

Total RNAs extracted from HeLa cells stably transfected with sh-Ctrl or sh-GRSF1 lentiviral plasmids were inspected by NanoDrop 2000 and Agilent Bioanalyzer 2100 first, then the qualified samples were subjected to the Affymetrix GeneChip Human Clariom™ D (cat#902923) for gene chip experiment at GeneChem company at Shanghai, China. GeneChip Scanner 3000 was used for chip scanning. The quality of the original chip data was evaluated first, and then the qualified data was subjected to data filtering. The remaining data meeting the filtering criteria was subjected to subsequent information analysis, including significant difference analysis and functional analysis of differential genes, such as Gene Ontology (GO) analysis and KEGG & BioCarta pathway analysis.

### Viral infection and transfection

For lentiviral short hairpin RNA (shRNA) infection, HEK293T cells were seeded at 50–60% confluency and cotransfected with either pLKO.1-vector or target gene pLKO.1-shRNA with packaging plasmid (psPAX2) and envelope plasmid (pMD2.G) using Lipofectamine 2000 (Invitrogen). Medium was changed 6 h later. After 48 h, the viral particles were harvested and filtered through a 0.45 μm filter and then used to infect parental cells for 12 h. The stably infected cells were then selected using 2 μg/mL puromycin for 4–6 days.

Two independent shRNA sequences against GRSF1 are listed below:

shGRSF1#1: 5’-CCGGGUGCCUCUCUGCUGCCGCATTCTCGAGAAUGCGGCAGCAGAGAGGCACTTTTTG-3’

shGRSF1#2:

5’-CCGGGCCCAAGACAUUAUAAACUTTCTCGAGAAAGUUUAUAAUGUCU

UGGGCTTTTTG-3’

Adeno-associated virus 9 (AAV9) vector which was carried out TBG promoter to ensure specifically targeting and expressing in liver tissue was used to construct plasmids AAV9-shGRSF1 and AAV9-GRSF1 and then prepared corresponding viruses. All AAV9 virus preparation was commissioned by Guangzhou Paizhen Biotechnology Co., LTD. The viruses were injected into mice through the tail vein and targeted to liver tissue. A blood coagulation activity test was performed after 9 weeks of viral injection.

AAV-shGRSF1:

5’-CCCATGTCCAACATAGGTATATTCAAGAGATATACCTATGTTGGACATGGG TTTTTT-3’

For transient transfection, plasmids were transfected using PEI (Invitrogen) reagent following the manufacturer’s instructions. The indicated plasmids were mixed with serum-free medium and PEI, incubated for 15 min at room temperature and then added to the cells cultured in DMEM with FBS. After 6–8 h transfection, the medium was replaced with fresh medium with 10% FBS, and transfected cells were harvested 48 h later. siRNA transfections were carried out using Lipofectamine RNAiMAX reagent (Invitrogen, cat#13778-150) according to the manufacturer’s protocol for 72 h, and the final concentrations of siRNAs were 10 nM. The siRNA sequences against GRSF1 were:

siGRSF1#1: 5’- GUGCCUCUCUGCUGCCGCATT-3’

siGRSF1#2: 5’- GCCCAAGACAUUAUAAACUTT-3’

control siRNA: 5′-UUCUCCGAAC GUGUCACGU-3′

### Antibodies

Antibodies raised against the following proteins were used at the indicated concentrations for western blotting (WB): rabbit anti-FLAG antibody (Sigma, F7425; 1:5000 for WB); rabbit anti-GRSF1 antibody (Abcam, ab205531; 1:1000 for WB); rabbit anti-Fibrinogen alpha chain antibody (Abcam, ab92572; 1:1000 for WB); rabbit anti-Fibrinogen beta chain antibody (Abcam, ab189490; 1:1000 for WB); mouse anti-Fibrinogen gamma chain antibody (Abcam, ab119948; 1:1000 for WB); rabbit anti-β-actin antibody (ABclonal, AC026; 1:10000 for WB); mouse anti-GAPDH antibody (ABclonal, AC002; 1:10000 for WB).

### Western blotting

Cells were washed twice with ice-cold 1×PBS and then lysed in RIPA buffer (Applygen Technologies) containing protease inhibitor cocktail (Amresco). Cell lysates were then centrifuged for 15 min at 15,000×*g* at 4 °C, and the insoluble debris was discarded. Protein concentration was determined by using a BCA protein assay reagent (Pierce). Cell lysates (20–40 μg) were subjected to 8–15% SDS-PAGE and transferred to nitrocellulose membranes (Millipore). The membrane was blocked using 5% milk in TBST buffer at room temperature for 1 h. Primary antibodies were blotted using 5% milk or BSA in TBST, and incubated at 4 °C overnight. Secondary antibodies (Dylight 800, Goat Anti-Rabbit IgG (H+L) (EarthOx, E032820) or Goat Anti-Mouse IgG (H+L) (EarthOx, E032810)) were incubated for 1 h at room temperature in 5% milk/TBST. Then the signals were captured by the Odyssey system.

### RNA isolation and real-time qPCR

Total RNA was isolated using the RNAiso Plus from Takara (9108) according to the manufacturer’s description. Then, 2 μg total RNA was transcribed into cDNA with the Hifair III 1st strand cDNA synthesis supermix for qPCR (H2208010, Yeasen) and subsequently analyzed using specific primers for GRSF1, FGA, FGB and FGG mRNA, and ACTIN, GAPDH primer as endogenous controls. Quantitative Real-Time PCR was carried out with an ABI 7500 Real-Time PCR system (Thermo Fisher Scientific). Relative RNA expression levels were calculated by the 2^−ΔΔCt^ method.

### ELISA

Cell culture supernatant was measured in vitro by a microplate reader (TECAN) according to the experimental steps and methods in the instruction of the kit (Human FG ELISA Kit, E-EL-H6106).

Mice serum was measured in vitro by a microplate reader (TECAN) according to the experimental steps and methods in the instruction of the kit (Mouse FG ELISA Kit, SEA193Mu).

### Biotinylated RNA pulldown assay and RNA-IP assay

Biotinylated RNA pulldown assay and RNA-IP were performed as previously described [46].

Short biotinylated RNAs were synthesized from GenePharma Company. The pulldown materials were subsequently analyzed by Western blotting.

### Animal preparation

All animal care and experimental protocols for in vivo studies followed the guidelines of the animal care and use committee of Peking University and were approved by the Ethics Committee of Peking University (approval no. LA2022345). The sample size for the animal studies was calculated based on a survey of data from published research or preliminary studies. Negative control (NC) mice, GRSF1 overexpression mice (GRSF1^+^) and GRSF1 knockdown mice (GRSF1^−^) were constructed by injecting control AAV virus, GRSF1-AAV virus and shGRSF1-AAV virus into the tail vein. After 9 weeks of AAV infection, mice were weighed, anesthetized with avertin, collected cardiac blood, and then sacrificed by neck broken, followed by systemic circulation perfusion with PBS. The total tissue lysates of liver, heart, spleen, and kidney were extracted and subjected to WB for indicated proteins. The group of young animals and the group of old animals were randomized before treatment, respectively. Mice were treated in a blinded fashion when animal experiments were carried out. No mice were excluded from the statistical analysis.

### Tail bleeding time and clotting time

Bleeding time: Mice were weighed, anesthetized with chloroform, measured from the tip of the tail, cut off vertically with a scalpel in 5 mm, and immediately began to time, and sucked blood with filter paper every 30 s until the blood was completely coagulated. Record the time.

Clotting time: Take blood canthus, connect a drop of blood on a slide, and start a stopwatch. Drop the blood with a dry needle every 10 s until the needle can pick out the fibrin filaments. Record the time.

### Ferric chloride–induced carotid artery injury

Mice were anesthetized, and the right common carotid artery was exposed. A miniature Doppler flow probe (model 0.5VB; Transonic Systems, Ithaca, NY) was positioned around the artery, and a 1 × 2-mm^2^ strip of 1 M Whatman filter paper (Whatman International) soaked in 20% FeCl_3_ was applied to the adventitia of the artery for 3 min. The filter paper was then removed, and thrombus formation in the artery was monitored via the blood flow rate until complete occlusion (flow rate 0 mL/min).

### Blood coagulation activity test

Mice were weighed and anesthetized with avertin. Cardiac blood was collected with 3.2% sodium citrate for anticoagulation, plasma was obtained by centrifugation at 3000 rpm for 10 min. Then platelet aggregation and coagulation factor analyzer (steellex) were used to quantitatively determine different blood coagulation parameters.

Fibrinogen values: Platelet aggregation and coagulation factor analyzer (steellex) were used to quantitatively determine the content of Fibrinogen in plasma in vitro according to the experimental steps and methods described in the manual of the liquid Fib assay kit (Clauss method) (steellex, SS00420052).

Activated partial thromboplastin time (APTT): APTT was quantitatively determined in vitro by platelet aggregation and coagulation factor analyzer according to the experimental steps and methods in the instruction of activated partial thrombin time determination Kit (coagulation method) (steellex, SS00220002).

Prothrombin time (PT): According to the procedure and method in the instruction of the prothrombin time determination kit (coagulation method) (steellex, SS00120002), PT was quantitatively determined in vitro by platelet aggregation and coagulation factor analyzer.

Thrombin time (TT): TT was quantitatively measured in vitro by platelet aggregation and coagulation factor analyzer according to the experimental steps and methods in the instruction of thrombin time determination kit (coagulation method) (steellex, SS00320002).

### Liver function

Serum metabolites were measured in vitro by a Toshiba 120 Automatic Biochemical analyzer according to the experimental steps and methods in the instruction of the kit (Beijing XinChuangYuan Biotech Co., LTD.).

### Statistical analysis

In all experiments, data were presented as the mean ± SEM. Unless otherwise stated, Student’s *t*-test was used to analyze statistical differences between groups. Statistical analyses were carried out using GraphPad (version 6.01). A two-tailed *P*-value of less than 0.05 was considered significant. *P < 0.05, **P < 0.01, ***P < 0.001.

## Results

### GRSF1 modulates fibrinogen mRNA and protein levels

**Fig. 1:**
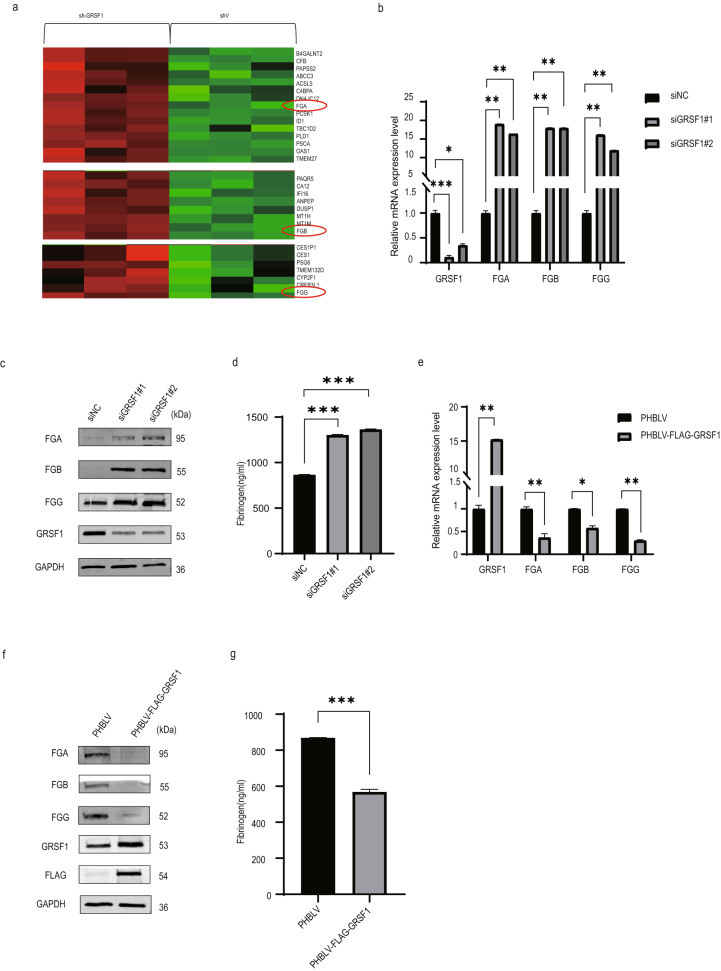
GRSF1 regulates fibrinogen expression at both mRNA and protein levels. **a** HeLa cells stably transfected with sh-Ctrl or sh-GRSF1 lentiviral plasmids were subjected to gene chip analysis. Gene chip results showed that mRNA levels of FGA, FGB and FGG (marked by red circle) increased after GRSF1 knockdown. **b**, **c** GRSF1 silencing increases fibrinogen gene expression. LO2 cells were transfected with siRNAs (siNC, siGRSF1#1 and siGRSF1#2), then total RNAs and whole cell lysates were extracted and subjected to RT-qPCR or WB analysis. GAPDH served as a loading control. **d** LO2 cells were treated as above. After 48 h, cell culture medium was collected and filtered for the ELISA experiment. **e**, **f** GRSF1 overexpression decreases fibrinogen gene expression. LO2 cells were transfected with PHBLV vector and PHBLV-GRSF1 plasmid, respectively. After 48 h, total RNAs and whole cell lysates were extracted and subjected to RT-qPCR or WB analysis. GAPDH served as a loading control. **g** LO2 cells were treated as above. After 48 h, cell culture medium was collected and filtered for ELISA experiment.

Only a few target RNAs regulated by GRSF1 were identified until now. In attempting to search for the novel target mRNAs of GRSF1, we performed gene chip analysis of sh-Ctrl and sh-GRSF1 in HeLa cells. Surprisingly, from gene chip results we found that mRNA levels of FGA, FGB and FGG, which encode Aα, Bβ and γ chain of fibrinogen, respectively, were upregulated by GRSF1 knockdown compared to sh-Ctrl samples (Fig. 1a).

To confirm gene chip results, GRSF1 was depleted by two independent siRNAs or shRNAs against GRSF1 in LO2 and HepG2 cells, respectively. Compared to vector control cells, GRSF1 knockdown remarkably upregulated FGA, FGB and FGG expressions at both mRNA and protein levels (Fig. 1b, c, and Supplementary Fig. S1A, B). Meanwhile, the secreted level of fibrinogen in supernatant detected by enzyme-linked immunoadsorbent assay (ELISA) was also increased (Fig. 1d, and Supplementary Fig. S1C). Conversely, GRSF1 overexpression significantly repressed their mRNA and protein levels (Fig. 1e, f, and Supplementary Fig. S1D, E), as well as fibrinogen secretory levels (Fig. 1g, and Supplementary Fig. S1F). Altogether, these results suggest that GRSF1 can simultaneously regulate FGA, FGB and FGG expressions at both mRNA and protein levels, as well as fibrinogen secretory level.

### GRSF1 decreases FGA, FGB, and FGG mRNA stability

**Fig. 2:**
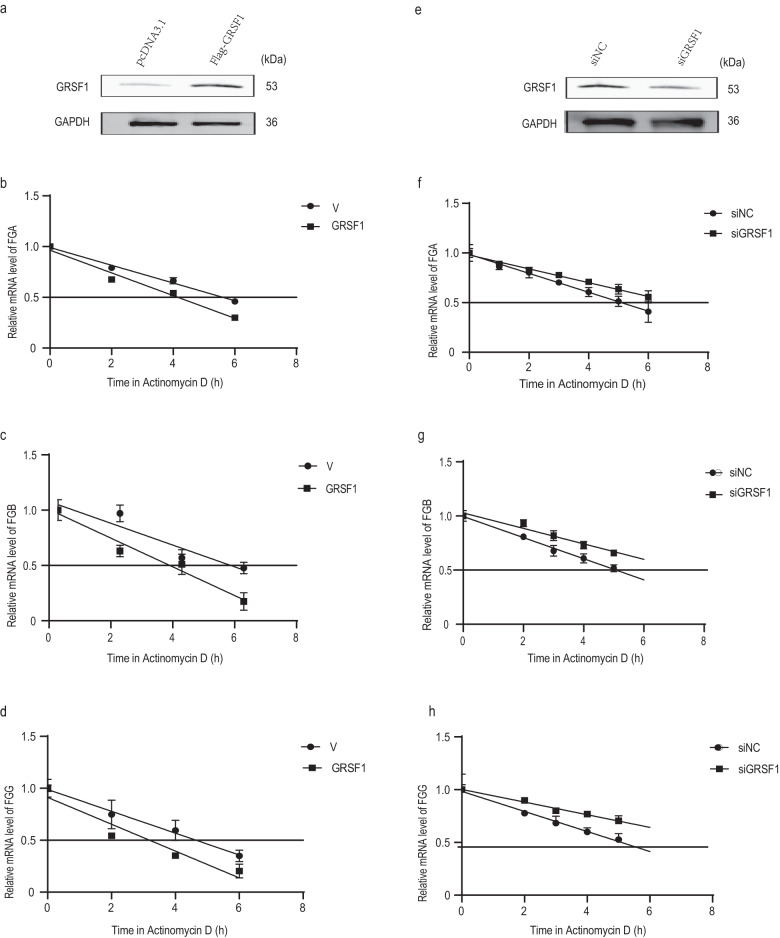
GRSF1 modulates fibrinogen mRNA stability. **a**–**d** GRSF1 overexpression reduces FGA, FGB, and FGG mRNAs half-life. 293T cells were transfected with pcDNA3.1 vector or pcDNA3.1-Flag-GRSF1 plasmid for 48 h and then exposed to Actinomycin D (2 μg/mL), and total RNAs were isolated at indicated times and subjected to real-time PCR to assess the half-life of FGA (**b**), FGB (**c**), and FGG (**d**) mRNAs. The half-life: GRSF1 OE vs Ctrl: (~4 h for FGA and FGB, ~3 h for FGG) vs (~5–6 h for FGA and FGB, ~4–5 h for FGG). **e**–**h** GRSF1 knockdown increases FGA, FGB, and FGG mRNAs half-life. 293T cells were transfected with siNC or siGRSF1, and then the half-life of FGA (**f**), FGB (**g**), and FGG (**h**) mRNAs were assessed by real-time PCR. The half-life: siGRSF1 vs siNC: (~7 h for FGA, FGB and FGG) vs (~5 h for FGA, FGB and FGG). Error bars in (**b**–**d**), and (**f**–**h**) represent as means ± SD from three independent experiments.

Given that GRSF1 is an RNA-binding protein, and GRSF1 could regulate FGA, FGB, and FGG expressions at both mRNA and protein levels (Fig. 1 and Supplementary Fig. S1), we speculated that GRSF1 might affect FGA, FGB and FGG mRNAs stability. To test this possibility, we assessed the effect of GRSF1 overexpression or knockdown on FGA, FGB and FGG mRNAs half-life in 293T cells, respectively. GRSF1 overexpression shortened FGA, FGB, and FGG mRNAs half-life (~4 h for FGA and FGB, ~3 h for FGG) compared to the vector control cells (~5–6 h for FGA and FGB, ~4–5 h for FGG) (Fig. 2a–d). Conversely, GRSF1 knockdown prolonged FGA, FGB, and FGG mRNAs half-life (~7 h for FGA, FGB, and FGG) compared with mock siRNA (~5 h for FGA, FGB, and FGG) (Fig. 2e–h). These results support the view that GRSF1 reduces FGA, FGB, and FGG mRNA stability to repress their expressions.

### GRSF1 interacts with CDS region of FGA, FGB and FGG mRNAs in vitro and in vivo

**Fig. 3:**
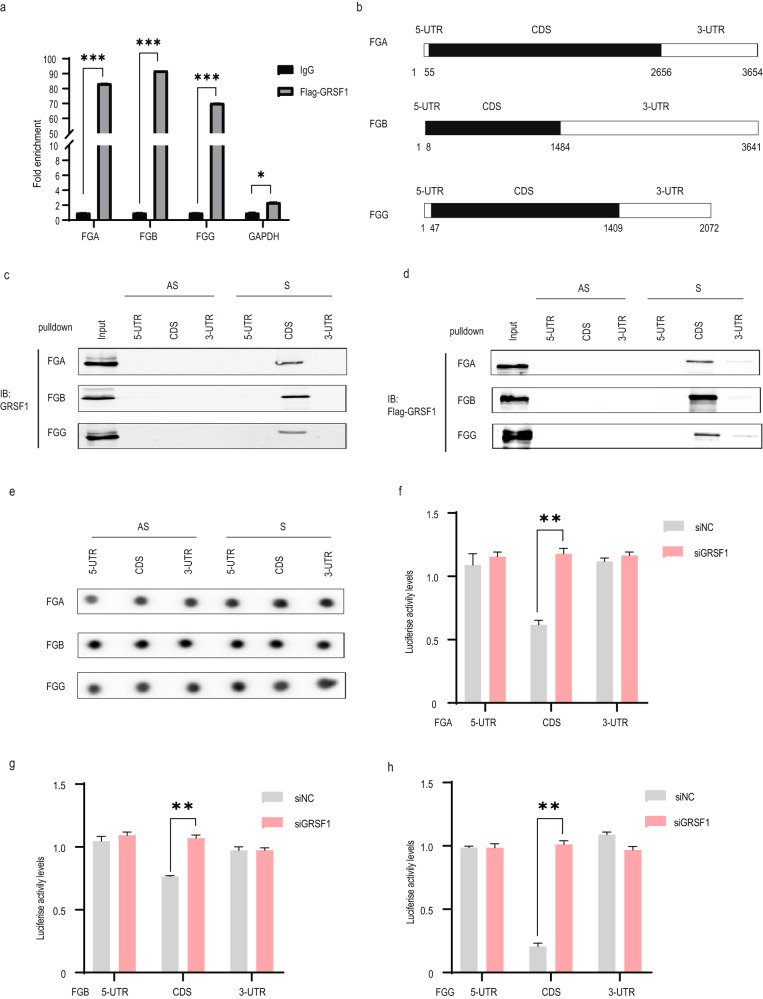
GRSF1 interacts with CDS region of fibrinogen three subunits mRNAs in vitro and in vivo. **a** Fibrinogen mRNA is immunoprecipitated by Flag-GRSF1. Total cell lysates from 293T cells transfected with pcDNA3.1 vector or pcDNA3.1-Flag-GRSF1 for 48 h were subjected to RNA-IP assays by incubating with anti-IgG or anti-Flag antibodies. The potential RNA-GRSF1 binding complexes were eluted and analyzed by real-time PCR specific for FGA, FGB and FGG mRNAs, respectively. Two pairs of primers were used for each of mRNAs. The enrichment for FGA, FGB, and FGG mRNAs was normalized to the vector. GAPDH mRNA was used as a nonspecific binding control. **b**–**e** GRSF1 binds to fibrinogen mRNA CDS. **b** Schematic representation of FGA, FGB and FGG mRNA 5’-UTR, CDS, and 3’-UTR. **c**, **d** Biotin-labeled fragments of 5’-UTR, CDS, and 3’-UTR of sense (S) or antisense (AS) of FGA, FGB and FGG mRNAs were amplified and subjected to biotin-labeled RNA-pulldown assay to detect bound GRSF1 or Flag-GRSF1 by WB. AS served as negative control. Input represented 1% of lysate used in pulldown reactions. **e** The amount of biotinylated mRNAs was detected by biotin antibody. **f**–**h** pGL3-derived luciferase reporter vectors bearing 5’-UTR, CDS, and 3’-UTR of FGA, FGB and FGG mRNAs were constructed and cotransfected with Renilla vector as well as with or without siRNA against GRSF1 in 293T cells, respectively. 48 h later, cell lysates were collected, and the luciferase activities against Renilla luciferase activities were measured by the double-luciferase assay system. Error bars represent as means ± SD from three independent experiments. *P < 0.05, **P < 0.01.

As an RBP, GRSF1 may bind to FGA, FGB and FGG mRNAs to affect their stability. To test this possibility, we carried out an RNA-IP assay by incubating anti-IgG or anti-Flag antibodies with total cell lysate from 293T cells transfected with Flag-GRSF1 plasmid. The potential Flag-GRSF1-RNA-binding complexes were eluted and analyzed by real-time PCR specific for FGA, FGB and FGG mRNAs, respectively. The results displayed that FGA, FGB and FGG mRNAs were remarkably enriched in Flag-IP samples compared with negative control IgG-IP samples (Fig. 3a). GAPDH mRNA was used as a nonspecific binding control. Negligible binding of GAPDH transcript with Flag-GRSF1 demonstrated that the interactions between FGA, FGB and FGG mRNAs with Flag-GRSF1 were specific.

To further confirm the interaction and to delineate the specific binding region of these mRNAs to GRSF1, we amplified biotin-labeled 5’-UTR, coding sequence (CDS), and 3’-UTR including both sense (S) and antisense (AS) sequences of FGA, FGB, and FGG mRNAs, respectively (Fig. 3b), and performed biotin-labeled RNA-pulldown assay. The results demonstrated that GRSF1 only interacted with biotinylated FGA, FGB and FGG mRNA S sequence CDS region in vivo and in vitro, instead of S sequence 5’-UTR or 3’-UTR, or entire AS (antisense) sequence (Fig. 3c, d). The similar biotin expression levels of all biotin-labeled RNA fragments were confirmed (Fig. 3e).

We further constructed luciferase reporter plasmids containing 5’-UTR, CDS, and 3’-UTR of FGA, FGB, and FGG mRNAs, respectively, and cotransfected them with Renilla vector and with or without siRNA against GRSF1, and then employed luciferase reporter gene assay. The luciferase activities of pGL3-CDS of FGA, FGB and FGG which can bind to GRSF1 significantly promoted by GRSF1 silencing, whereas the luciferase activities of pGL3-5’-UTR and pGL3-3’-UTR of FGA, FGB and FGG remained unchanged in the presence or absence of GRSF1 (Fig. 3f–h). Altogether, these results imply that GRSF1 is the FGA, FGB and FGG mRNAs-binding protein and GRSF1 binds to the CDS regions of these three mRNAs to reduce their stability, thereby repressing their expression levels.

### The G-tracts of fibrinogen C region within FGA, FGB and FGG mRNAs CDS and qRRM2 domain of GRSF1 are required for their interaction

**Fig. 4:**
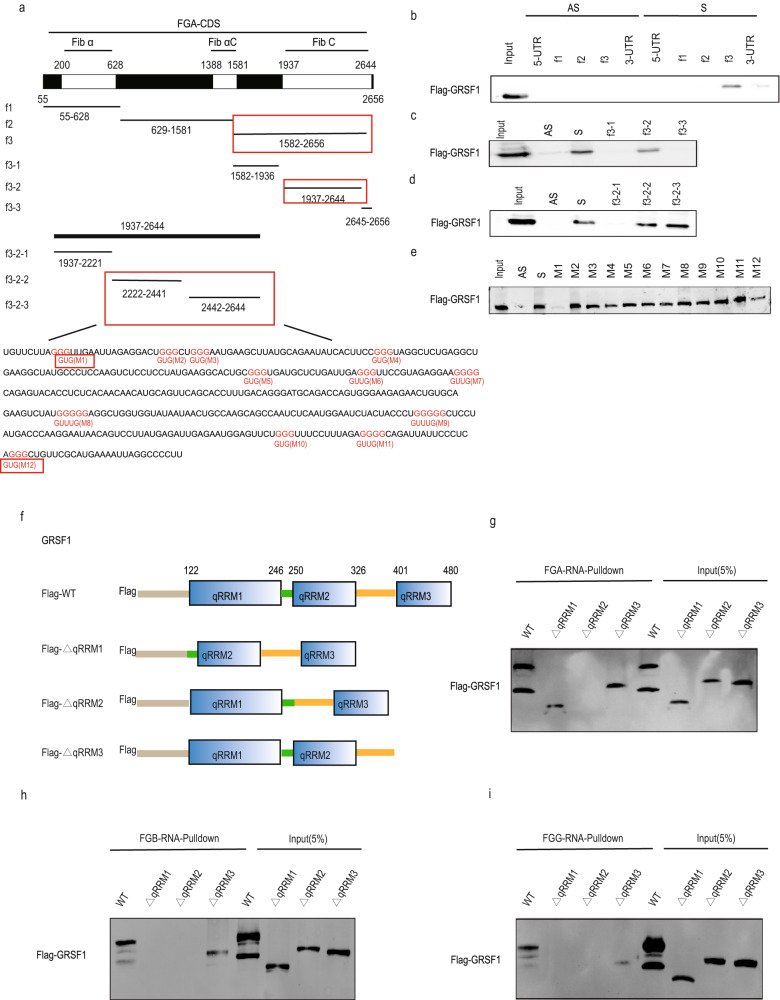
The G-tracts of fibrinogen C region within FGA, FGB, and FGG mRNAs CDS and qRRM2 domain of GRSF1 are required for their interaction. **a**–**e** Identification of the specific binding region of FGA mRNA to GRSF1. **a** Upper panel: schematic diagram of FGA mRNA CDS. Middle panel: different fragments of FGA mRNA CDS. Lower panel: nucleotide sequences of 2222–2644 fragment G to U mutants. G to U substitutions made in this fragment are marked in red. **b**–**e** Different biotinylated RNA fragments were subjected to RNA-pulldown assay to detect bound proteins by WB. **f** Schematic representation of three qRRM domains of GRSF1, as well as their truncations lacking qRRM1, qRRM2, or qRRM3 domain, respectively. **g**–**i** Biotin RNA-pulldown assays were performed as aforementioned method, and the binding of FGA, FGB, and FGG mRNA to each GRSF1 truncation was detected by WB. 5% of whole cell extracts used as input.

To further narrow down the specific binding region between FGA, FGB and FGG mRNAs CDS regions with GRSF1, we first split the full length of FGA mRNA CDS into three fragments with each containing Fib α, Fib αC, and Fib C domain, respectively (Fig. 4a, upper panel), and performed RNA-pulldown assay. The result demonstrated that FGA mRNA CDS fragment 3 (f3) containing Fib C domain was the main binding region of GRSF1 instead of other two fragments (Fig. 4b). Similarly, GRSF1 mainly associated with FGB fragment 2 (f2) and FGG fragment 2 (f2) both containing Fib C domain (Supplementary Figs. S3A and S4A, upper panel; and Supplementary Figs. S3B and S4B).

We then split FGA f3 into three parts to further define the binding region of FGA mRNA CDS with GRSF1 (Fig. 4a, middle panel). GRSF1 interacted with f3-2 fragment (Fib C domain), but not with f3-1 and f3-3 fragments absence of Fib C domain (Fig. 4c). Similar to FGA, GRSF1 associated with FGB f2-2 fragment (Fib C domain) and FGG f2-2 fragment (Fib C domain), but not with other two fragments without Fib C domain, respectively (Supplementary Figs. S3A and S4A, middle panel; and Supplementary Figs. S3C and S4C). Together, these results suggest that Fib C domain of FGA, FGB and FGG mRNAs CDS is bona fide required for the binding to GRSF1.

GRSF1 possesses three qRRM domains which preferentially bind to G-rich RNA sequences (29–32). FGA f3-2 fragment contains multiple G-tracts that may be the binding site of GRSF1. According to the distribution of G-tracts, f3-2 fragment was further divided into three segments (Fig. 4a, lower panel). The RNA-pulldown results displayed that f3-2-2 and f3-2-3 fragments (2222–2644) were the main binding regions of GRSF1 (Fig. 4d). Similarly, FGB f2-2 fragment and FGG f2-2 fragment were also further divided into two segments, respectively (Supplementary Figs. S3A and S4A, lower panel), and FGB f2-2-1 (719–1083) segment and FGG f2-2-2 (894–1293) segment were the main binding regions of GRSF1 (Supplementary Figs. S3D and S4D).

FGA 2222–2644 fragment contains twelve G-tracts that are possible to bind to GRSF1. To further investigate the contribution of each G-tract in association with GRSF1, we substituted the middle G to U in each of the twelve G-tracts, respectively (named as M1-M12, Fig. 4a, lower panel, labeled as red color). The RNA-pulldown result showed that the binding ability of M1 mutant to GRSF1 almost completely diminished and M12 mutant binding activity significantly decreased compared to FGA CDS 2222–2644 sense fragment (Fig. 4e). Conversely, M2-M11 mutants preserved substantial binding capability to GRSF1 (Fig. 4e). Similarly, four G to U mutants within FGB f2-2-1 fragment and three G to U mutants within FGG f2-2-2 fragment (Supplementary Figs. S3A and S4A, lower panel, labeled as red color) results showed that FGB M2 mutant and FGG M1 and M2 mutants almost completely lost the binding ability to GRSF1, FGG M3 mutant possessed weak binding activity, whereas the other three mutants of FGB reserved the similar binding activity to GRSF1 as FGB f2-2-1 fragment (Supplementary Figs. S3E and S4E). Collectively, these data suggest that only a few G-tracts within the Fib C domain of FGA, FGB and FGG mRNAs CDS are important for the interaction with GRSF1, especially the first G-tract of FGA, the second G-tract of FGB, and the first and second G-tracts of FGG, are bona fide required for the binding to GRSF1.

We further constructed luciferase reporter plasmids containing every fragment and mutants of FGA, FGB and FGG mRNAs CDS which are depicted in Fig. 4a, Supplementary Figs. S3A and S4A, respectively, to prove the minimal sequence for GRSF1 binding. Compared to siNC samples, GRSF1 knockdown only significantly augmented the luciferase activities of those fragments that possessed the binding ability to GRSF1, which is displayed in Supplementary Figs. S2A–C, S3F–H, and S4F–H, but had no effect on other fragments without binding activity to GRSF1. Moreover, GRSF1 deletion also only significantly elevated the luciferase activities of the corresponding wildtype mRNA fragments of FGA, FGB and FGG, but had no effect on those G-tract mutants that lost the binding ability to GRSF1, which displayed in Supplementary Figs. S2D, S3I and S4I. Altogether, these results are consistent with the corresponding RNA-pulldown results (Fig. 4a–e, Supplementary Figs. S3A–E and S4A–E), which demonstrate that GRSF1 regulates all of these fragments and mutants expression levels depending on its binding capacity to them.

GRSF1 contains three qRRM motifs (qRRM1-3) that are responsible for the binding to G-rich RNA sequence. To identify the GRSF1 domain requirement for FGA, FGB, and FGG mRNAs CDS interaction, we generated the individual qRRM motif truncated mutants of GRSF1 (Fig. 4f) and performed RNA-pulldown assay. Compared to wildtype (WT) GRSF1, GRSF1-∆qRRM1 truncation preserved partial binding activity to FGA CDS, whereas completely lost binding ability to FGB and FGG CDS (Fig. 4g–i, 2nd lane). GRSF1-∆qRRM2 truncation completely lost binding ability to FGA, FGB, and FGG mRNAs CDS (Fig. 4g–i, 3rd lane). GRSF1-∆qRRM3 truncation preserved some extent of binding activity to FGA, FGB, and FGG mRNAs CDS (Fig. 4g–i, 4th lane). Collectively, these data imply that the qRRM2 domain is bona fide required for GRSF1 binding to all FGA, FGB, and FGG mRNAs CDS, and the qRRM1 domain is also critical for GRSF1 binding to FGB and FGG mRNAs CDS.

### GRSF1 expression decreases, while fibrinogen expression and blood coagulation activity increase in old mice

The underlying mechanism of increased plasma fibrinogen level and hypercoagulability during human and animal aging is still elusive. GRSF1 expression level decreases during cell and animal aging [42–45]. Here we demonstrated that GRSF1 could reduce FGA, FGB, and FGG mRNAs stability to repress their expressions and fibrinogen secretory level (Figs. 1 and 2, and Supplementary Fig. S1). Moreover, the KEGG pathway analysis result from our gene chip data suggested that GRSF1 might regulate the blood coagulation pathway (Supplementary Fig. S5A). These findings prompted us to hypothesize whether GRSF1 could negatively regulate plasma fibrinogen level thereby antagonizing blood coagulation activity and reducing hypercoagulability in old mice.

**Fig. 5:**
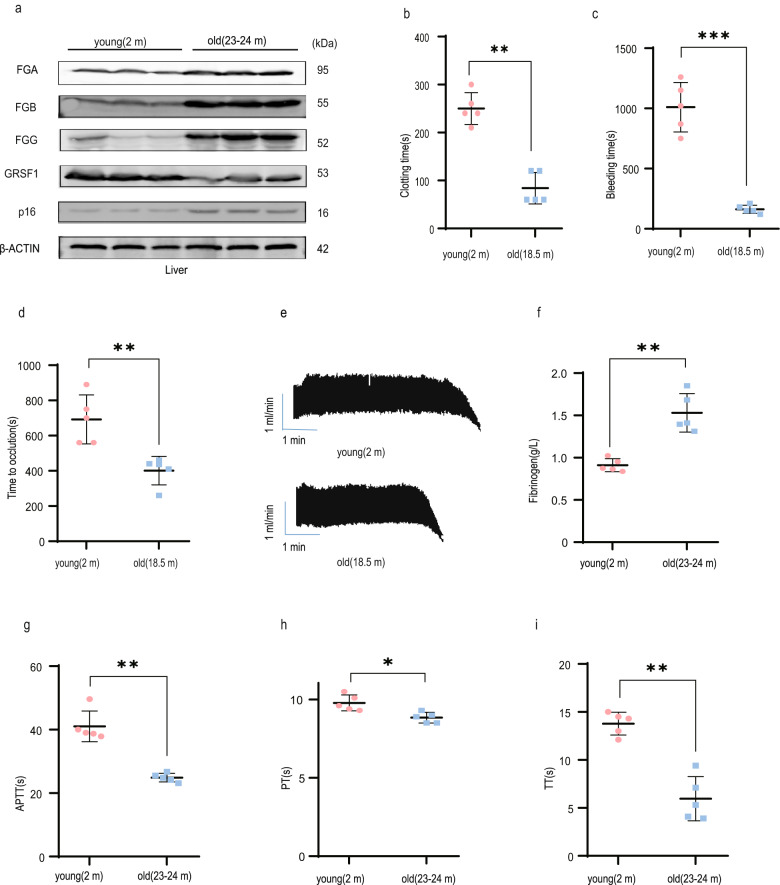
GRSF1 expression decreases, while fibrinogen expression and blood coagulation activity increase, during mice aging. **a** GRSF1 expression level decreases in old mice liver. The total tissue lysates of liver from young and old C57/BL6 male mice were extracted and subjected to WB for indicated proteins. **b** Blood clotting time in young and old mice. **c** Tail bleeding time in young and old mice. **d**, **e** Representative Doppler echocardiogram of the blood flow rate in mouse right carotid arteries following FeCl_3_ injury (**d**). The time when the flow rate was reduced to 0 mL/min was considered as the complete occlusion time. Statistic results of the right carotid artery complete occlusion time (**e**). **f**–**i** Serum fibrinogen concentration (**f**), APTT, PT, and TT of the platelet-free plasma isolated from young and old C57/BL6 male mice were measured by using corresponding kits. *P < 0.05, **P < 0.01, and ***P < 0.001.

To explore this hypothesis, we first examined GRSF1 and fibrinogen expression levels in young (2 months) and old (23–24 months) C57/BL6 male mice (*n* = 5 per group). Consistent with previous reports [42–45], GRSF1 protein and mRNA levels in liver in old male mice significantly decreased compared to young mice. Conversely, FGA, FGB and FGG expression levels in old mice liver substantially increased (Fig. 5a, and Supplementary Fig. S5B). The senescent state of old liver was confirmed by p16 upregulation. Similar results were obtained in liver in young (2 months) and old (38–42 months) BALB/c female mice (Supplementary Fig. S6A, B) (*n* = 5 per group). Agreed with previous studies, we also observed that GRSF1 expression levels in muscle, brain, heart, spleen and kidney notably downregulated in old mice compared to young mice (Supplementary Figs. S5C–E and S6C).

To confirm age-associated hypercoagulability, we first compared the clotting time and tail bleeding time between young (2 months) and old (18.5 months) C57/BL6 male mice (*n* = 5 per group). Both clotting time and tail bleeding time of old mice significantly shortened compared with young mice (Fig. 5b, c). To further investigate the effects of age on thrombus formation in vivo, we performed FeCl_3_-induced thrombosis assay using mouse carotid arteries. The time required for vessel occlusion was significantly reduced in old mice compared with young mice (Fig. 5d, e). Similarly, the clotting time and tail-bleeding time as well as the time of occlusion of old (18–20 months) BALB/c female mice also significantly shortened relative to young mice (2 months) (*n* = 5 per group) (Supplementary Fig. S6D–G). Altogether, these results confirm the hypercoagulability during mice aging.

Age-associated hypercoagulability is accompanied by the increased plasma concentration of multiple coagulation factors [1–3], therefore we further compared fibrinogen plasma level and multiple liver-synthesized coagulation factors mRNA levels between young (2 months) and old (23–24 months) C57 male mice. Compared to young mice, the fibrinogen plasma concentration significantly increased (Fig. 5f), and mRNA levels of multiple coagulation factors such as F7, F8, F9, F12, F13 elevated in old mice (Supplementary Fig. S5F). Similarly, the fibrinogen plasma concentration largely increased, and multiple coagulation factors mRNA levels elevated in old (38–42 months) BALB/c female mice compared to young mice (Supplementary Fig. S6H, L).

We also measured the mRNA levels of several other liver-produced proteins including anticoagulant proteins antithrombin and Protein C, and the fibrinolytic system proteins including plasminogen and α_2_-antiplasmin (α_2_-AP), which are all involved in the coagulation pathway. Compared to young mice, the mRNA levels of antithrombin and Protein C displayed the increase trend or significantly elevated in old mice, whereas plasminogen and α_2_-AP levels showed no significant difference between young and old mice (Supplementary Figs. S5G and S6M).

We further assessed the plasma coagulation activity by measuring activated partial thromboplastin time (APTT), prothrombin time (PT), and thrombin time (TT) between young (2 months) and old (23–24 months) C57/BL6 male mice. Compared to young mice, the APTT, PT and TT values in old mice significantly reduced (Fig. 5g–i) (*n* = 5 per group), which suggested that the plasma coagulation activity substantially enhanced in old mice versus young mice. Similarly, the APTT, PT, and TT values in old BALB/c female mice (38–42 months) all significantly reduced compared to young mice (2 months) (Supplementary Fig. S6I–K) (*n* = 5 per group). Overall, our results suggest that although the levels of some anticoagulant proteins such as antithrombin and Protein C also elevate during mice aging to try to counteract the age-related increase of multiple coagulation factors, however, the net effect of coagulation activity versus anticoagulation activity is towards the hypercoagulability during mice aging, which is proved by the decrease of clotting time, tail-bleeding time, the time of occlusion, APTT, PT, and TT values in old mice compared to young mice.

### GRSF1 overexpression decreases fibrinogen plasma level and mitigates blood coagulation activity in old mice

**Fig. 6:**
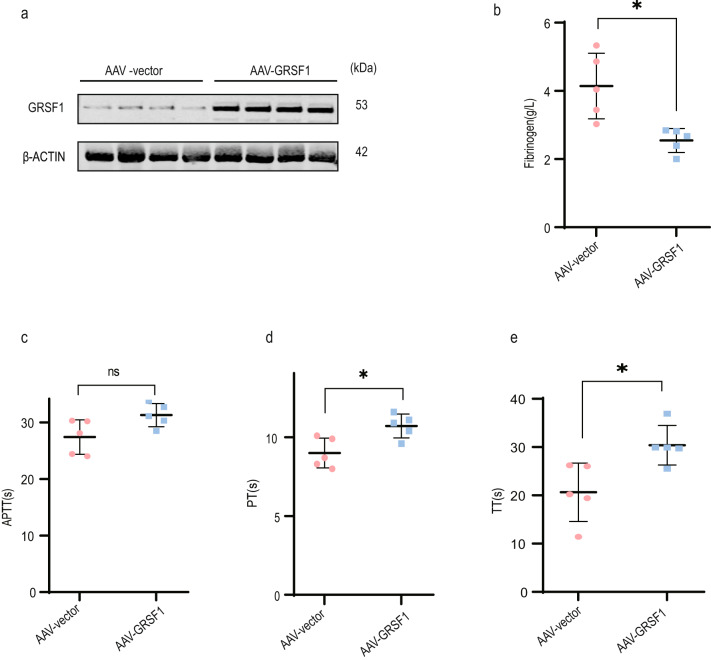
GRSF1 overexpression decreases fibrinogen plasma level and attenuates blood coagulation activity in old mice. **a** Old BALB/c female mice (19–26 months) were infected by the indicated AAV9 virus, respectively. After 9 weeks of AAV9 infection, the GRSF1 expression level in mice liver was analyzed by WB. **b**–**e** GRSF1 overexpression decreases fibrinogen plasma level and blood coagulation activity in old mice. Fibrinogen plasma concentration (**b**), APTT (**c**), PT (**d**), and TT (**e**) values were determined.

To investigate whether GRSF1 could regulate plasma fibrinogen level thereby antagonizing blood coagulation activity in vivo, first, we used adeno-associated virus 9 (AAV9) which was carried out TBG promoter to ensure specifically targeting and expressing in liver tissue to infect old BALB/c female mice (19–26 months). Mice were injected with AAV vector virus or AAV-GRSF1 virus (*n* = 5 per group), respectively. After 9 weeks of AAV infection, the GRSF1 overexpression effect in mice liver was confirmed at both mRNA and protein levels (Fig. 6a and Supplementary Fig. S7A). Consistent with cell culture results, we found that GRSF1 overexpression in mice liver also significantly repressed FGA, FGB, and FGG mRNA levels (Supplementary Fig. S7A).

We then determined the effect of GRSF1 overexpression on blood coagulation activity in these mice. Compared to AAV mice, the fibrinogen plasma concentration of AAV-GRSF1 mice detected either by Clauss method on Steellex analyzer or by ELISA kit significantly decreased or displayed the decreased trend (Fig. 6b, and Supplementary Fig. S7B). Moreover, the APTT value of AAV-GRSF1 mice displayed the increased trend relative to AAV mice, although it did not reach a significant difference (Fig. 6c), whereas the PT and TT values of AAV-GRSF1 mice significantly elevated compared with AAV mice (Fig. 6d, e). Meanwhile, GRSF1 overexpression in old mice liver did not change the mRNA levels of multiple coagulation factors, anticoagulant proteins, and fibrinolytic system proteins synthesized in liver such as F7, F8, F9, F10, F11, F12, F13, antithrombin, Protein C, thrombopoietin (TPO), plasminogen, and α_2_-AP compared to AAV mice (Supplementary Fig. S7C, D). Altogether, these results imply that AAV9-mediated GRSF1 overexpression in aged mice liver specifically mitigates fibrinogen plasma level and may reduce age-associated hypercoagulability without exerting an off-target effect on other blood factors.

Fibrinogen and many blood coagulation factors are synthesized in the liver [14–18], and AAV9 infection might cause damage to the liver, which in turn would affect fibrinogen and coagulation factors synthesis and concentrations. To exclude this possibility, we detected liver weight/body weight ratio, and liver function indexes including alanine aminotransferase (ALT), aspartate aminotransferase (AST), total protein (TP), albumin (ALB), and alkaline phosphatase (ALP). All of these indexes in AAV-GRSF1 mice showed no significant difference compared to AAV mice (Supplementary Fig. S7E). These data indicate that AAV-mediated GRSF1 overexpression does not cause liver damage.

### GRSF1 knockdown increases fibrinogen plasma level and promotes blood coagulation activity in young mice

**Fig. 7:**
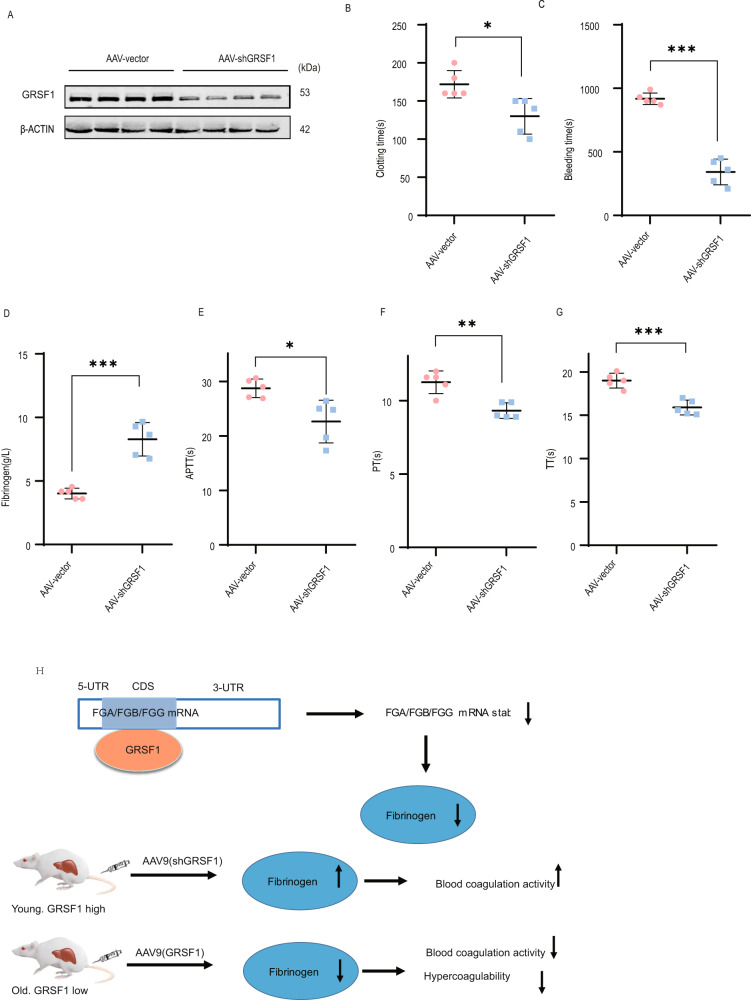
GRSF1 knockdown increases fibrinogen plasma level and promotes blood coagulation activity in young mice. **A** Two-months young BALB/c mice were infected by the indicated AAV9 virus, respectively. After 9 weeks of AAV9 infection, the GRSF1 expression level in mice liver was analyzed by WB. **B**, **C** After 8.5 weeks of AAV9 infection, the clotting time (**B**) and tail bleeding time (**C**) were measured. **D**–**G** GRSF1 knockdown increases fibrinogen plasma level and blood coagulation activity in young mice. Fibrinogen plasma concentration (**D**), APTT (**E**), PT (**F**), and TT (**G**) values were determined. **H** Schematic model of GRSF1 regulates fibrinogen expression and blood coagulation activity.

We further used AAV9 to infect young BALB/c mice (2 months). Mice were injected with AAV empty vector virus (negative control, AAV-vector), or AAV-shGRSF1 virus (AAV-shGRSF1) (*n* = 5 per group), respectively. After 9 weeks of AAV infection, the GRSF1 knockdown effect in mice liver was confirmed at both mRNA and protein levels (Fig. 7A, and Supplementary Fig. S8A). Consistent with cell culture results, we observed that GRSF1 knockdown in mice liver also significantly upregulated FGA, FGB, and FGG mRNA levels (Supplementary Fig. S8A).

To investigate whether GRSF1 knockdown has an effect on hemostasis, we first analyzed the tail bleeding time and clotting time after 8.5 weeks of AAV9 infection. Both clotting time and tail bleeding time of AAV-shGRSF1 mice significantly shortened compared to AAV mice (Fig. 7B, C). These data indicate that GRSF1 knockdown in young mice liver accelerates hemostasis.

We further examined plasma fibrinogen level and blood coagulation activity after 9 weeks of AAV9 infection. Compared to AAV mice, the fibrinogen plasma concentration of AAV-shGRSF1 mice detected either by Clauss method or by ELISA all significantly increased (Fig. 7D, and Supplementary Fig. S8B). Furthermore, the APTT, PT and TT values of AAV-shGRSF1 mice all significantly reduced compared to AAV mice (Fig. 7E–G). Meanwhile, GRSF1 knockdown in young mice liver also did not change the mRNA levels of multiple coagulation factors, anticoagulant proteins, TPO, and fibrinolytic system proteins compared to AAV mice (Supplementary Fig. S8C, D). Collectively, these results demonstrate that AAV9-mediated GRSF1 knockdown in young mice liver specifically upregulates plasma fibrinogen level and promotes blood coagulation activity without exerting an off-target effect on other blood factors.

The liver weight/body weight ratio and all liver function indexes in AAV and AAV-shGRSF1 mice showed no significant difference (Supplementary Fig. S8E), which indicate that AAV-mediated GRSF1 knockdown does not cause liver damage.

## Discussion

In this study, we found that GRSF1 could antagonize the age-associated hypercoagulability via posttranscriptional regulation of fibrinogen expression, and revealed GRSF1 as an endogenous negative regulator of fibrinogen plasma level and blood coagulation activity. The elevated fibrinogen plasma level during aging may be driven by age-associated chronic, low-grade inflammatory state, in which IL-6 plasma level increases [25, 26, 47, 48]. Accelerated epigenetic aging characterized by DNA methylation age may also increase fibrinogen gene transcription [49]. However, the underlying mechanism is still elusive. Here, we proved that GRSF1 could negatively regulate fibrinogen plasma level and blood coagulation activity in vivo. GRSF1 overexpression in old mice liver specifically decreased fibrinogen plasma level and attenuated hypercoagulability in vivo without affecting other blood factors (Fig. 6). Therefore, the reduction of hypercoagulability is solely attributed to the GRSF1 overexpression specifically suppresses fibrinogen expression in old mice liver. Similarly, GRSF1 knockdown in young mice liver specifically increased plasma fibrinogen level and augmented blood coagulation activity in vivo without affecting other blood factors (Fig. 7). Altogether, we suggest that the decrease of GRSF1 expression level in the liver during animal aging at least partially contributes to the increase of fibrinogen plasma level and hypercoagulability (Fig. 7H). Hence, our findings provide a new explanation for why fibrinogen plasma level increases with animal age.

Our study revealed that GRSF1 negatively regulated fibrinogen gene expression both in vitro and in vivo, thus antagonizing fibrinogen plasma level and blood coagulation activity in vivo (Fig. 7H). Fibrinogen is the most abundant blood coagulation factor and plays a critical role in both primary and secondary hemostasis. The elevated plasma fibrinogen level is an independent risk factor for atherosclerosis, myocardial infarction, heart failure, ischemic stroke, and vein thrombosis [20–23], and it may also be one of the reasons for the increased incidence of such diseases in the elderly people [9–13]. For these reasons, our findings have important significance. Our results not only unveil the important role of GRSF1 in regulating blood coagulation activity but also imply that the decrease of GRSF1 expression level during animal and human aging might increase the risk of such disease incidence by upregulating fibrinogen plasma level. Moreover, our findings also suggest that GRSF1 might be a novel diagnostic marker or even a new target for the prevention or treatment of age-associated hypercoagulability, cardiovascular, cerebrovascular, and thrombotic diseases. However, this idea needs to be further explored in the future.

GRSF1 participates in mitochondrial RNA metabolism, erythropoiesis, viral infection, cell senescence [33–45], etc., and its function in the blood coagulation process has never been reported. Here, we found that GRSF1 could antagonize blood coagulation activity in vivo, which suggests that GRSF1 may implicate in hemostasis and blood coagulation process via modulation of fibrinogen gene expression (Figs. 6 and 7). Therefore, our findings identify fibrinogen as the novel target of GRSF1 and unveil the novel function of GRSF1 in the blood coagulation process and hemostasis, to our knowledge for the first time.

Fibrinogen gene expression is regulated at transcriptional, epigenetic, and posttranscriptional levels, as well as the IL-6-STAT3 axis [25–28]. However, the regulatory mechanism of fibrinogen gene expression, especially its posttranscriptional level regulation is barely understood. Here, we identified that GRSF1 as an RNA-binding protein could simultaneously bind to fibrinogen three subunits FGA, FGB, and FGG mRNAs CDS region (Fig. 3) and decreased their mRNAs stability (Fig. 2), thereby reducing fibrinogen expression level and secretory level (Fig. 1). Therefore, our study shed new light on the posttranscriptional regulation of fibrinogen by GRSF1.

We found that GRSF1 could interact with fibrinogen all three chains FGA, FGB, and FGG mRNAs CDS regions (Fig. 3), and the Fib C domain within FGA, FGB, and FGG mRNAs CDS was bona fide required for the binding to GRSF1 (Fig. 4, and Supplementary Figs. S2–4). We further identified that only a few G-tracts within the Fib C domain were important for FGA, FGB, and FGG mRNAs CDS association with GRSF1 (Fig. 4, and Supplementary Figs. S2–4), which agreed with previous studies that GRSF1 preferentially binds to G-rich RNA sequences [29–32]. Additionally, the qRRM2 domain of GRSF1 was bona fide required for GRSF1 binding to FGA, FGB, and FGG mRNAs CDS, and qRRM1 domain was also critical for GRSF1 binding to FGB and FGG mRNAs CDS (Fig. 4f–i), which was consistent with previous findings that qRRMs of GRSF1 may act in concert [31]. Therefore, we elucidate the detailed binding mechanism between GRSF1 and fibrinogen mRNAs, and these results suggest a uniform posttranscriptional regulatory mechanism in which GRSF1 regulates fibrinogen expression.

In summary, we report here that GRSF1 negatively regulates fibrinogen gene expression at posttranscriptional level in vitro and in vivo, thus antagonizing fibrinogen plasma level and blood coagulation activity in vivo.

### Supplementary information

Supplementary File

Original Data File

## Data Availability

The datasets and materials obtained during the current study are available from the corresponding authors upon reasonable request.
