# Supplementary material for: GRSF1 antagonizes age-associated hypercoagulability via modulation of fibrinogen mRNA stability
Source: Cell Death Dis. 2023 Nov 3;14(11):717. doi: 10.1038/s41419-023-06242-9 (PMC10624831; doi:10.1038/s41419-023-06242-9)
Supplement: Supplementary file 1 — Supplementary File [file 41419_2023_6242_MOESM1_ESM.docx]

**GRSF1 antagonizes age-associated hypercoagulability** **via modulation of fibrinogen mRNA stability**

Doudou Liu^1^, Chenzhong Xu^2^, Ze Gong^3, 4^, Yijie Zhao^5^, Zhiqiang Fang^1^, Xiaoli Rao^1^, Qingyu Chen^1^, Guodong Li^1^, Tanjun Tong^1^, Wei Kong^3,^*, Jun Chen^1,^*

**Supplemental Materials and methods**

**RNA isolation and real-time qPCR**

The following primer pairs were used for Real-Time-PCR:

Human:

GRSF1-F: 5′-GGCTGCGGCTGTAACTG-3′

GRSF1-R: 5′-CCGACGGGATAGAGCCA-3′

FGA-F: 5’-ATGAAACGACTGGAGGTGG-3’

FGA-R: 5’-TACTTCACGAGCTAAAGCCC-3’

FGB-F: 5’-GTGTCTGGCAAAGAATGTG-3’

FGB-R: 5’-AGAACTGTCAGGTTGAATGAG-3’

FGG-F: 5’-GTGTAGCATATGTTGCTACCAG-3’

FGG-R: 5’-ATGCCACAGGTAGTTGGAC-3’

ACTIN-F: 5′-CCAACCGCGAGAAGATGA-3′

ACTIN-R: 5′-TCCATCACGATG CCAGTG-3′

GAPDH-F: 5’-TGCACCACCAACTGCTTAGC-3’

GAPDH-R: 5’-GGCATGGACTGTGGTCATGAG-3’

Mouse:

GRSF1-F: 5’-TATCTCATTCGAGCTCAGGG-3’

GRSF1-R: 5’-TCGGATTCTGCAGTCTGAG-3’

FGA-F: 5’-AAGCGCAACAGATTCAAGC-3’

FGA-R: 5’-TATCAATATCCACCTCCAGGC-3’

FGB-F: 5’-AACAGAACCATGACCATCC-3’

FGB-R: 5’-TGGATCCGTAGTTACCCAG-3’

FGG-F: 5’-GCTAAGCAGCAGTTCTTAGTG-3’

FGG-R: 5’-GCCATCAATCCTCTTCTGC-3’

β-actin-F: 5’-ATATCGCTGCGCTGGTCGTC-3’

β-actin-R: 5’-AGGATGGCGTGAGGGAGAGC-3’

F7-F: 5’-GATCTTCAAGAGCCCTGAGAG-3’

F7-R: 5’-AGCAGACGTAAGACTTGAGATG-3’

F8-F: 5’-GTCAGTCCTCTACATGCAAGG-3’

F8-R: 5’-ACCGTGGATCTGATTTAGTTGG-3’

F9-F: 5’-GTGTGAATCAAATCCTTGTTTAAATGG-3’

F9-R: 5’-TTACAAAACTGCTTGCACCTG-3’

F10-F: 5’-AGAAGACGAAAGAATACTGGACC-3’

F10-R: 5’-TTGCCTTCAAATCCCTCCG-3’

F11-F: 5’-CAGGATGACCTCATTACATCAGG-3’

F11-R: 5’-ACCAAGCGGCAGTATGTG-3’

F12-F: 5’-CTGGATATTTTTGCGACTTGGAC-3’

F12-R: 5’-CTTGCTTTTCAGTCATGTTCCG-3’

F13-F: 5’-TCGGGAAGCTAATTGTGACC-3’

F13-R: 5’-ACATCAGAGCAGTTTCAAGGG-3’

Antithrombin-F: 5’-TCAAAGATGTCATCCCACAGG-3’

Antithrombin-R: 5’-GACTGCCCATCGACCTTATAG-3’

Protein C-F: 5’-AAGGATAGTCAACGGAACGC-3’

Protein C-R: 5’-CAGGAAGTGTGGATGAGCA-3’

Plasminogen-F: 5’-AGAATGTAAGACCGGCATCG-3’

Plasminogen-R: 5’-CATTGGGATGTGTACTGGGAG-3’

α-AP-F: 5’-ACTGACCTGTTCTCTTTGGTG-3’

α-AP-R: 5’-GCTGTGTAGTGTTTGGTTCTG-3’

TPO-F: 5’-AGCATGGACCTTGAACTATGG-3’

TPO-R: 5’-TGGAAACAGGAGTATACAAGCAC-3’

**Biotinylated RNA pull down assay and RNA-IP assay**

To prepare templates for in vitro transcription, the following primer pairs were used:

T7: 5’- CCAAGCTTCTAATACGACTCACTATAGGGAGA

FGA-5’UTR-sense: 5’-(T7) aatcctttctttcagctggagtgctcctcaggagccagccccacccttagaaaag

FGA-5’UTR-antisense: 5’-(T7) cttttctaagggtggggctggctcctgaggagcactccagctgaaagaaa ggatt

FGA-CDS-sense: (T7) atgttttccatgaggatcgtctgcc

FGA-CDS-antisense: (T7) ctattgggtcacaaggggcctaattttc

FGA-3’UTR-sense: (T7) gctgaagaagtgggaatgggagc

FGA-3’UTR-antisense: (T7) ttgcttgggatattttaatacatattcaagagaaaatgacaaatatatc

FGB-5’UTR-sense: (T7) gacagtgctgacactacaaggctcggagctccgggcactcagacatc

FGB-5’UTR-antisense: (T7) gatgtctgagtgcccggagctccgagccttgtagtgtcagcactgtc

FGB-CDS-sense: (T7) atgaaaaggatggtttcttggagcttcc

FGB-CDS-antisense: (T7) ctattgctgtgggaagaagggcc

FGB-3’UTR-sense: (T7) tccccaatacgtagatttttgctcttctgtatg

FGB-3’UTR-antisense: (T7) ttttggaggtgatggatatatttaatatcttgactgtgc

FGG-5’UTR-sense: (T7) gacagtgctg acactacaaggctcggagctccgggcactcagacatc

FGG-5’UTR-antisense: (T7) gatgtctgagtgcccggagctccgagccttgtagtgtcagcactgtc

FGG-CDS-sense: (T7) atgagttggtccttgcacccc

FGG-CDS-antisense: (T7) ctacaaatcatcctcagggtaaagtgagtc

FGG-3’UTR-sense: (T7) aaaattaactgctaacttctattgacccacaaagtttc

FGG-3’UTR-antisense: (T7) gtagataaattatcatcagcataaaactgttatggagttttcaac

FGA-55-sense: (T7) atgttttccatgaggatcgtctgcc

FGA-55-antisense: (T7) ttcacgagctaaagccctactgc

FGA-629-sense: (T7) gtagatctgaaggactatgaagatcagcagaag

FGA-629-antisense: (T7) gaacccatccagagtacctatgcc

FGA-1582-sense: (T7) cgccataggcaccctgatg

FGA-1582-antisense: (T7) ctattgggtcacaaggggcctaattttc

FGA-1937-sense: (T7) cctgtcagagactgtgatgatgtcctc

FGA-1937-antisense: (T7) aaggggcctaattttcatgcgaacag

FGA-2645-sense: (T7) gtgacccaatag

FGA-2645-antisense: (T7) ctattgggtcac

FGA-2222-sense: (T7) tgttcttagggttgaattagaggactggg

FGA-2222-antisense: (T7) ccactggtctgcatccctgtc

FGA-2442-sense: (T7) gaagagaactgtgcagaagtctatgggg

FGA-2442-antisense: (T7) aaggggcctaattttcatgcgaacag

FGA-M1-sense: (T7) tgttcttag**a**gttgaattagaggactgggctgggaatg

FGA-M2-sense: (T7) tgttcttagggttgaattagaggactg**a**gctgggaatg

FGA-M3-sense: (T7) tgttcttagggttgaattagaggactgggctg**a**gaatg

FGA-M4-sense: (T7) tgttcttagggttgaattagaggactgggctgggaatgaagcttatgcagaatatcacttcc g**a**gtag

FGA-M5-sense-1-F: (T7) tgttcttagggttgaattagaggactggg

FGA-M5-sense-1-R: gagcatcac**t**cgcagtgcc

FGA-M5-sense-2-F: ggcactgcg**a**gtgatgctc

FGA-M5-sense-2-R: aaggggcctaattttcatgcgaacag

FGA-M6-sense-1-F: (T7) tgttcttagggttgaattagaggactggg

FGA-M6-sense-1-R: gtactctgc**tt**cttcctctacgg

FGA-M6-sense-2-F: ccgtagaggaag**aa**gcagagtac

FGA-M6-sense-2-R: aaggggcctaattttcatgcgaacag

FGA-M7-sense-1-F: (T7) tgttcttagggttgaattagaggactggg

FGA-M7-sense-1-R: ggtctgcatc**t**ctgtcaaaggtg

FGA-M7-sense-2-F: cacctttgacag**a**gatgcagacc

FGA-M7-sense-2-R: aaggggcctaattttcatgcgaacag

FGA-M8-sense-1-F: (T7) tgttcttagggttgaattagaggactggg

FGA-M8-sense-1-R: caccagcctc**ttt**catagacttc

FGA-M8-sense-2-F: gaagtctatg**aaa**gaggctggtg

FGA-M8-sense-2-R: aaggggcctaattttcatgcgaacag

FGA-M9-sense-1-F: (T7) tgttcttagggttgaattagaggactggg

FGA-M9-sense-1-R: gtcataggagc**ttt**cagggtagtag

FGA-M9-sense-2-F: ctactaccctg**aaa**gctcctatgac

FGA-M9-sense-2-R: aaggggcctaattttcatgcgaacag

FGA-M10-sense-F:

(T7) tgttcttagggttgaattagaggactgggctgggaatgaagcttatgcagaatatcacttccgggtag

FGA-M10-sense-R: aaggggcctaattttcatgcgaacagccctgagggaataatctgcccctctaaaggaaac**t**cag

FGA-M11-sense-F:

(T7) tgttcttagggttgaattagaggactgggctgggaatgaagcttatgcagaatatcacttccgggtag

FGA-M11-sense-R:

aaggggcctaattttcatgcgaacagccctgagggaataatctgc**tt**ctctaaag

FGA-M12-sense-F:

(T7) tgttcttagggttgaattagaggactgggctgggaatgaagcttatgcagaatatcacttccgggtag

FGA-M12-sense-R:

aaggggcctaattttcatgcgaacagctctgagggaataatctgcccctctaaag

FGB-9-sense: (T7) atgaaaaggatggtttcttggagcttcc

FGB-9-antisense: (T7) aggaatattgcaactgacagtgcatg

FGB-712-sense: (T7) gtggtgtctggcaaagaatgtgagg

FGB-712-antisense: (T7) ctattgctgtgggaagaagggcc

FGB-719-sense: (T7) tctggcaaagaatgtgaggaaattatcagg

FGB-719-antisense: (T7) gaagggcctgatcttcatactcatcttcc

FGB-1471-sense: (T7) ttcccacagcaatag

FGB-1471-antisense: (T7) ctattgctgtgggaa

FGB-1084-sense: (T7) gattcactgtacagaatgaagccaacaaatacc

FGB-1084-antisense: (T7) gaagggcctgatcttcatactcatcttcc

FGB-M1-sense-1-F: (T7) tctggcaaagaatgtgaggaaattatcagg

FGB-M1-sense-1-R: **a**catttcctgccaaagtcaacactacc

FGB-M1-sense-2-F: ggtagtgttgactttggcaggaaatg**a**

FGB-M1-sense-2-R: ctccatagtgagcctttactttgtctcc

FGB-M2-sense-1-F: (T7) tctggcaaagaatgtgaggaaattatcagg

FGB-M2-sense-1-R: **t**ctgtttatatggatcccatttcctgcc

FGB-M2-sense-2-F: ggcaggaaatgggatccatataaacag**a**

FGB-M2-sense-2-R: ctccatagtgagcctttactttgtctcc

FGB-M3-sense-1-F: (T7) tctggcaaagaatgtgaggaaattatcagg

FGB-M3-sense-1-R: **t**catctgtgttggttgcaacatttcc

FGB-M3-sense-2-F: ggaaatgttgcaaccaacacagatg**a**

FGB-M3-sense-2-R: ctccatagtgagcctttactttgtctcc

FGB-M4-sense-1-F: (T7) tctggcaaagaatgtgaggaaattatcagg

FGB-M4-sense-1-R: **t**catcctggtaagctggctaattttatcatttc

FGB-M4-sense-2-F: gaaatgataaaattagccagcttaccaggatg**a**gacccacagaacttttgatagaaatggaggactggaaaggagacaaagtaaaggctcactatggag

FGB-M4-sense-2-R: ctccatagtgagcctttactttgtctcctttccagtcctccatttctatcaaaagttctgtgggtc**t**catcctggtaagctggctaattttatcatttc

FGG-48-sense: (T7) atgagttggtccttgcacccc

FGG-48-antisense: (T7) atggatttgcaccgtgtctttgc

FGG-565-sense: (T7) gatatcactgggaaagattgtcaagacattgc

FGG-565-antisense: (T7) ctacaaatcatcctcagggtaaagtgagtc

FGG-572-sense: (T7) actgggaaagattgtcaagacattgcc

FGG-572-antisense: (T7) gaatgggattatcttcatagtggttttcttcatgg

FGG-1294-sense: (T7) aacagactcacaattggagaaggacag

FGG-1294-antisense: (T7) ctacaaatcatcctcagggtaaagtgagtc

FGG-894-sense: (T7) ggcatagtctgcagtactggttct

FGG-894-antisense: (T7) gaatgggattatcttcatagtggttttcttcatgg

FGG-M1-sense-F: (T7) agaaccagtactgcagactatgccatgttcaaggtg**a**gacctg

FGG-M1-sense-R: gaatgggattatcttcatagtggttttcttcatggaataccaccgggttttc

FGG-M2-sense-1-F: (T7) agaaccagtactgcagactatgcc

FGG-M2-sense-1-R: catctccagcatc**tt**caccag

FGG-M2-sense-2-F: ctggtg**aa**gatgctggagatg

FGG-M2-sense-2-R: ccatgaagaaaaccactatgaagataatcccattc

FGG-M3-sense-F: (T7) agaaccagtactgcagactatgccatgttcaaggtgggacctg

FGG-M3-sense-R: gaatgggattatcttcatagtggttttcttcatggaataccaccgggttttccaagtggc**t**caaataatg

**Supplementary Figures**


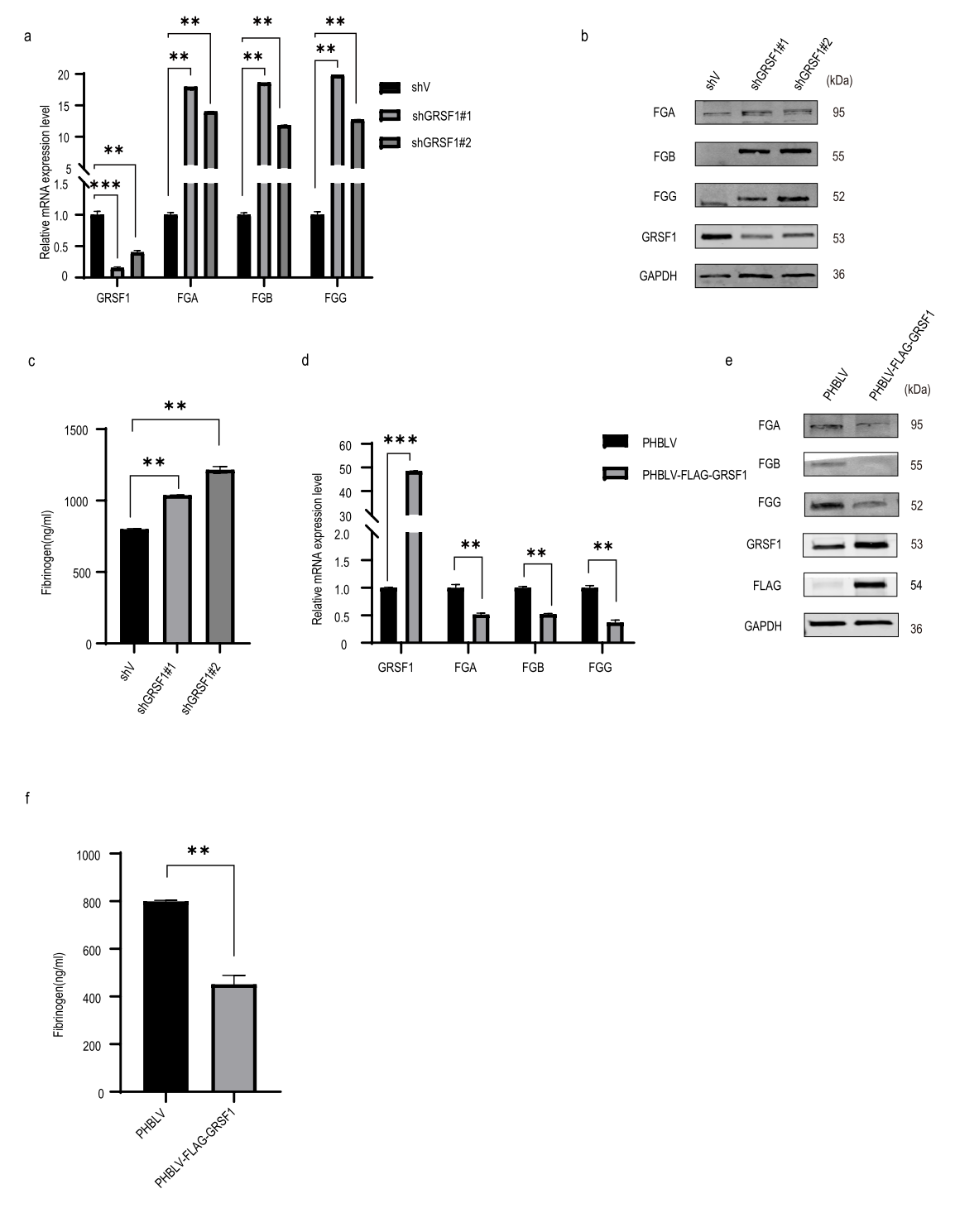


**Fig. S1** **GRSF1 regulates fibrinogen mRNA and protein levels.** **(related to Fig. 1)**

(A-C) GRSF1 silencing increases fibrinogen gene expression and secretory level. HepG2 cells were stably transfected with shV, shGRSF1#1 and shGRSF1#2 lentiviral plasmids, then total RNAs and whole cell lysates were extracted and subjected to RT-qPCR (A), or WB analysis (B). GAPDH served as a loading control. Cell culture medium was collected and filtered for ELISA detection (C). (D-F) GRSF1 overexpression decreases fibrinogen gene expression and secretory level. HepG2 cells were transfected with PHBLV vector and PHBLV-GRSF1 plasmid, respectively. After 48 hours, total RNAs and whole cell lysates were extracted and subjected to RT-qPCR (D) or WB analysis (E). GAPDH served as a loading control. Cell culture medium was collected and filtered for ELISA detection (F).


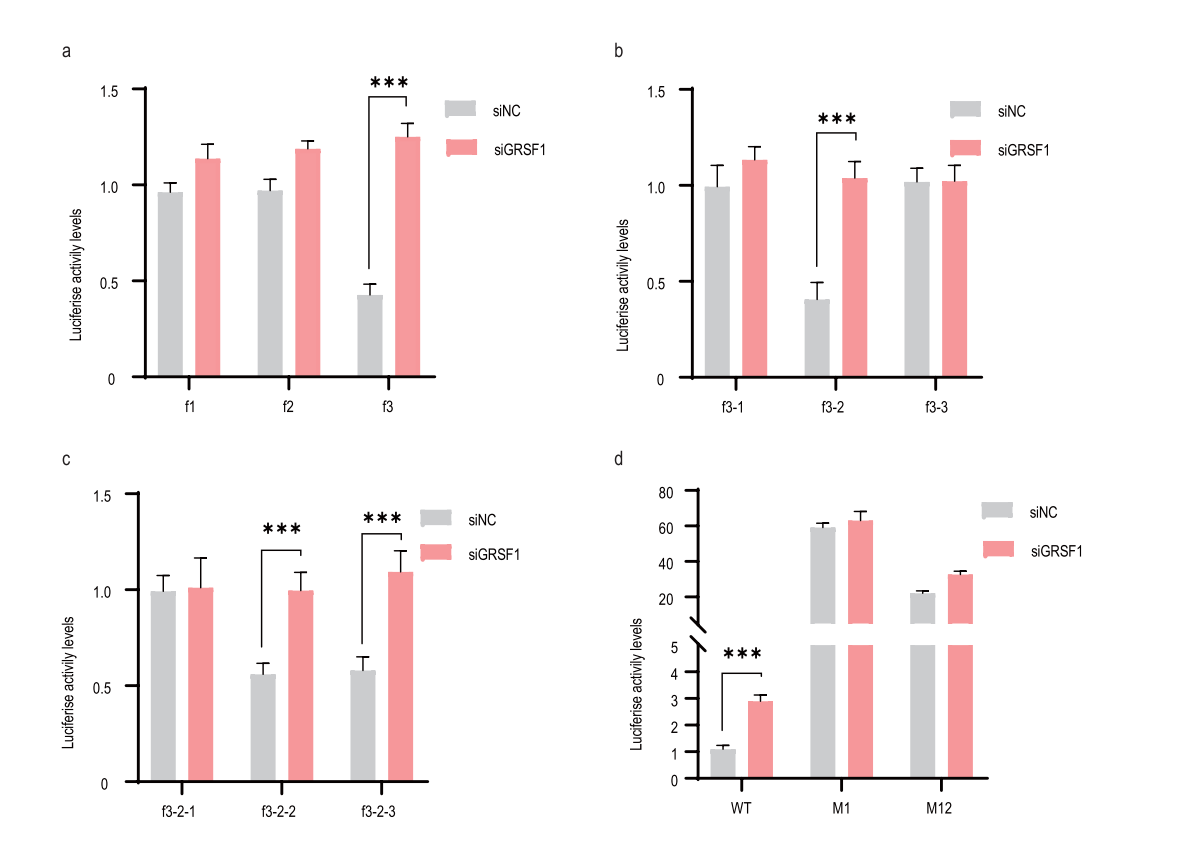


**Fig. S2 GRSF1 regulates FGA mRNA CDS fragments and mutants expression depending on its binding capacity to these fragments and mutants (related to Fig. 4).**

(A-D) pGL3-derived luciferase reporter vectors bearing different fragments and mutants of FGA mRNA CDS were constructed and cotransfected with Renilla vector as well as with or without siRNA against GRSF1 in 293T cells, respectively. 48 h later, cell lysates were collected, and the luciferase activities against Renilla luciferase activities were measured by the double-luciferase assay system. Error bars represent as means ± SD from three independent experiments. *P < 0.05, **P < 0.01, and ***P< 0.001.


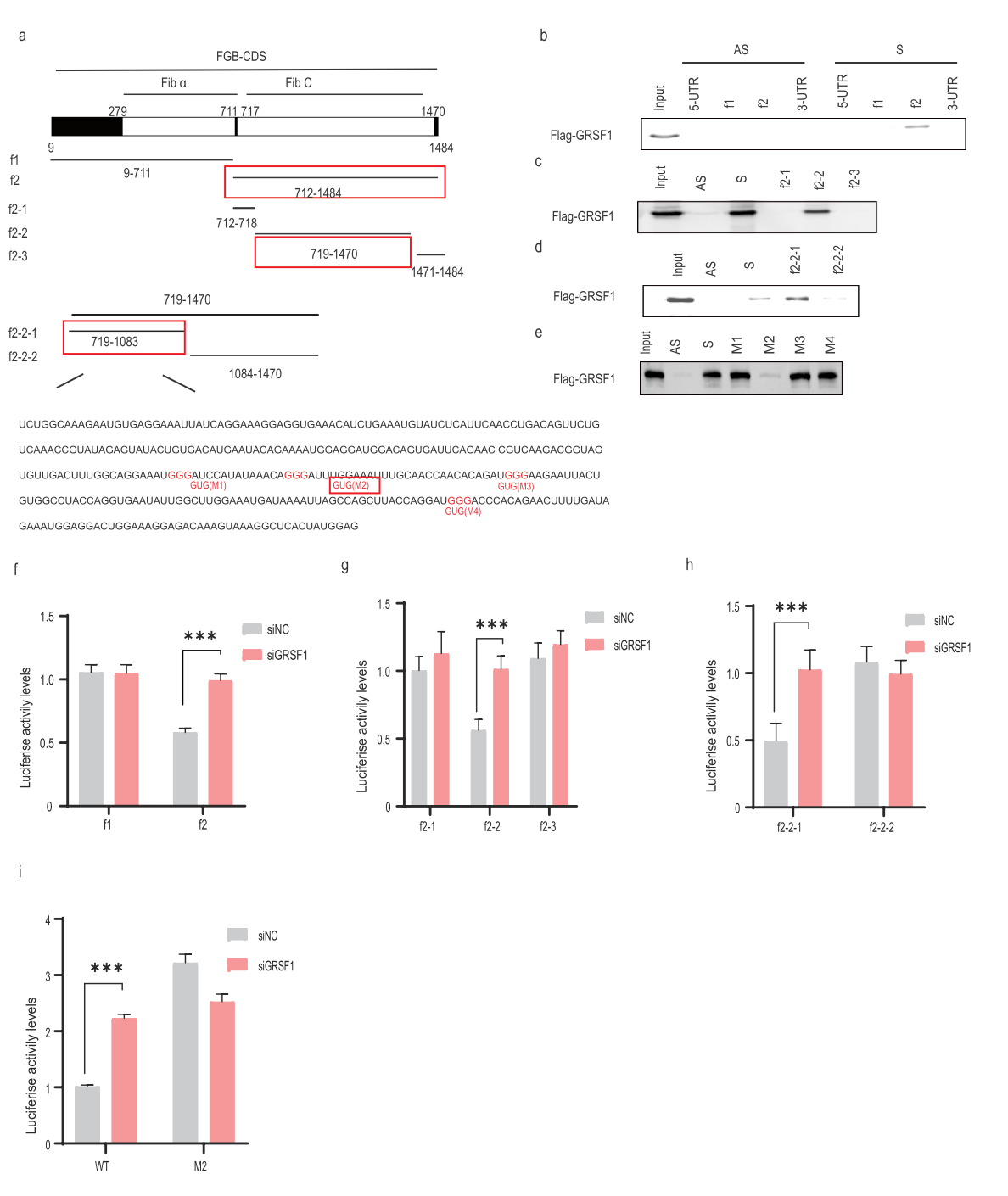


**Fig. S3** **The G-tract of fibrinogen C region within FGB mRNA CDS is required for FGB mRNA-GRSF1 interaction (related to Fig. 4).**

(A-E) Identification of the specific binding region of FGB mRNA to GRSF1. (A) Upper panel: schematic diagram of FGB mRNA CDS. Middle panel: different fragments of FGB mRNA CDS. Lower panel: nucleotide sequences of 719-1083 fragment G to U mutants. G to U substitutions made in this fragment are marked by red. (B-E) Different biotinylated RNA fragments were subjected to RNA pulldown assay to detect bound proteins by WB. (F-I) pGL3-derived luciferase reporter vectors bearing different fragments and mutants of FGB mRNA CDS were subjected to the double-luciferase reporter assay. Error bars represent as means ± SD from three independent experiments. *P < 0.05, **P < 0.01, and ***P< 0.001.


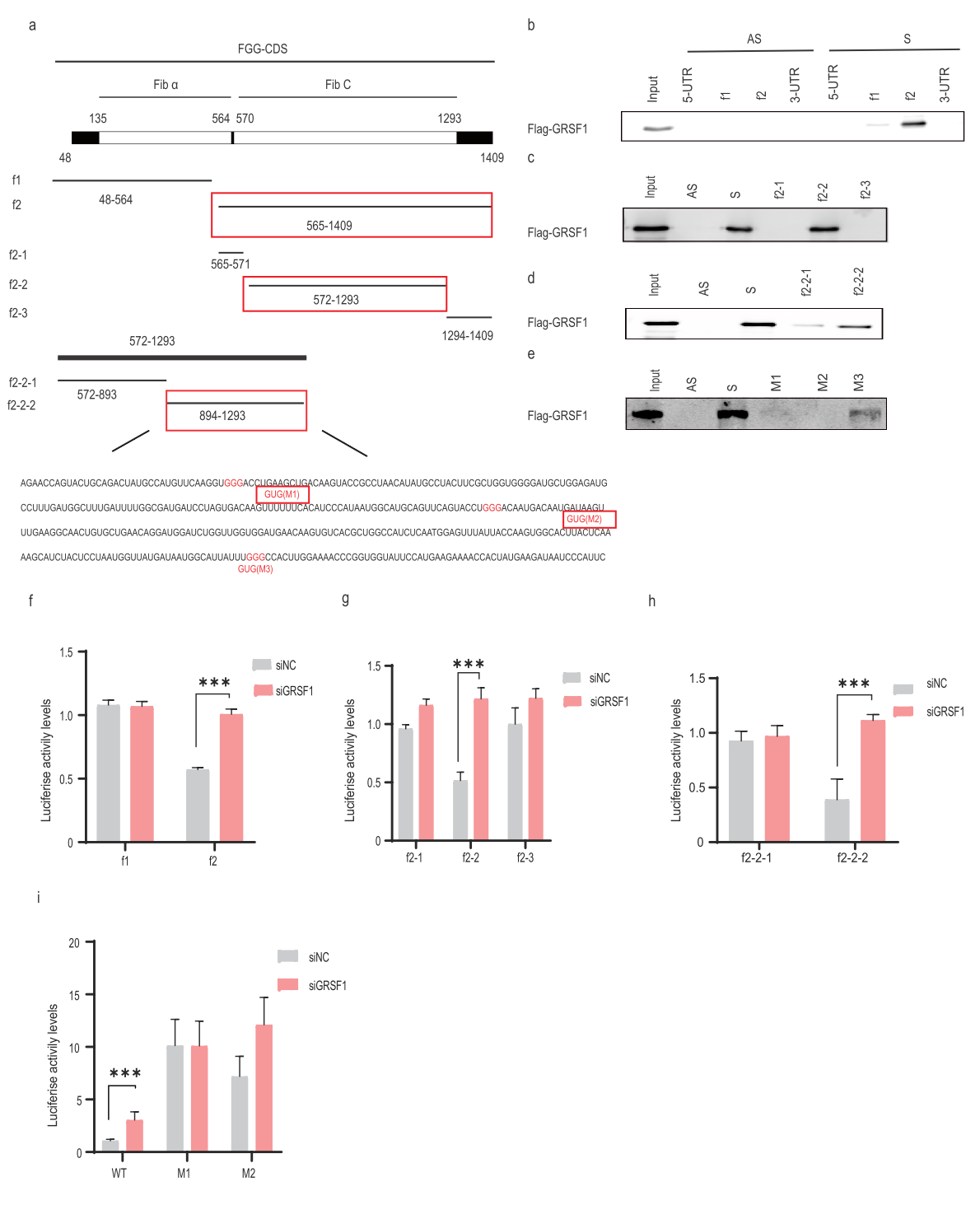


**Fig. S4** **The G-tract of fibrinogen C region within FGG mRNA CDS is required for FGG mRNA-GRSF1 interaction (related to Fig. 4).**

(A-E) Identification of the specific binding region of FGG mRNA to GRSF1. (A) Upper panel: schematic diagram of FGG mRNA CDS. Middle panel: different fragments of FGG mRNA CDS. Lower panel: nucleotide sequences of 894-1293 fragment G to U mutants. G to U substitutions made in this fragment are marked by red. (B-E) Different biotinylated RNA fragments were subjected to RNA pulldown assay to detect bound proteins by WB. (F-I) pGL3-derived luciferase reporter vectors bearing different fragments and mutants of FGG mRNA CDS were subjected to the double-luciferase reporter assay. Error bars represent as means ± SD from three independent experiments. *P < 0.05, **P < 0.01, and ***P< 0.001.


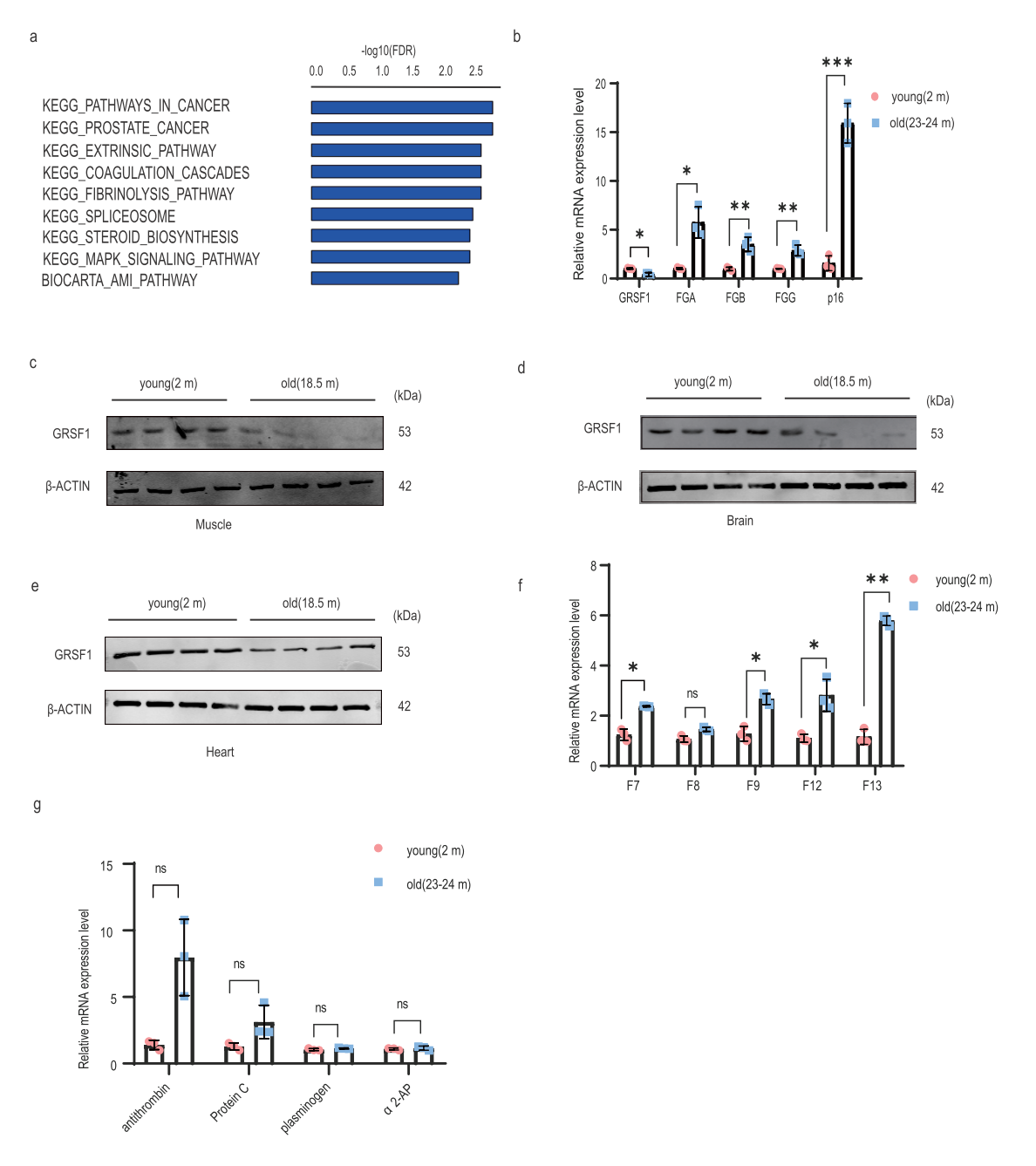


**Fig. S5 GRSF1 expression level decreases in multiple tissues, while many liver-produced coagulation factors mRNA levels increase, during C57/BL6 male mice aging. (related to Fig. 5)**

(A) The KEGG pathway analysis result from gene chip data. (B) The total RNAs of liver from young and old C57/BL6 male mice were extracted and subjected to RT-qPCR analysis. (C-E) GRSF1 expression levels decrease in multiple tissues in old mice. The total tissue lysates of muscle, brain, and heart from young and old C57/BL6 male mice were extracted and subjected to WB analysis. (F and G) The total RNAs of liver from young and old C57/BL6 male mice were extracted and subjected to RT-qPCR analysis for multiple liver-produced coagulation factors, anticoagulant proteins, and fibrinolytic system proteins.


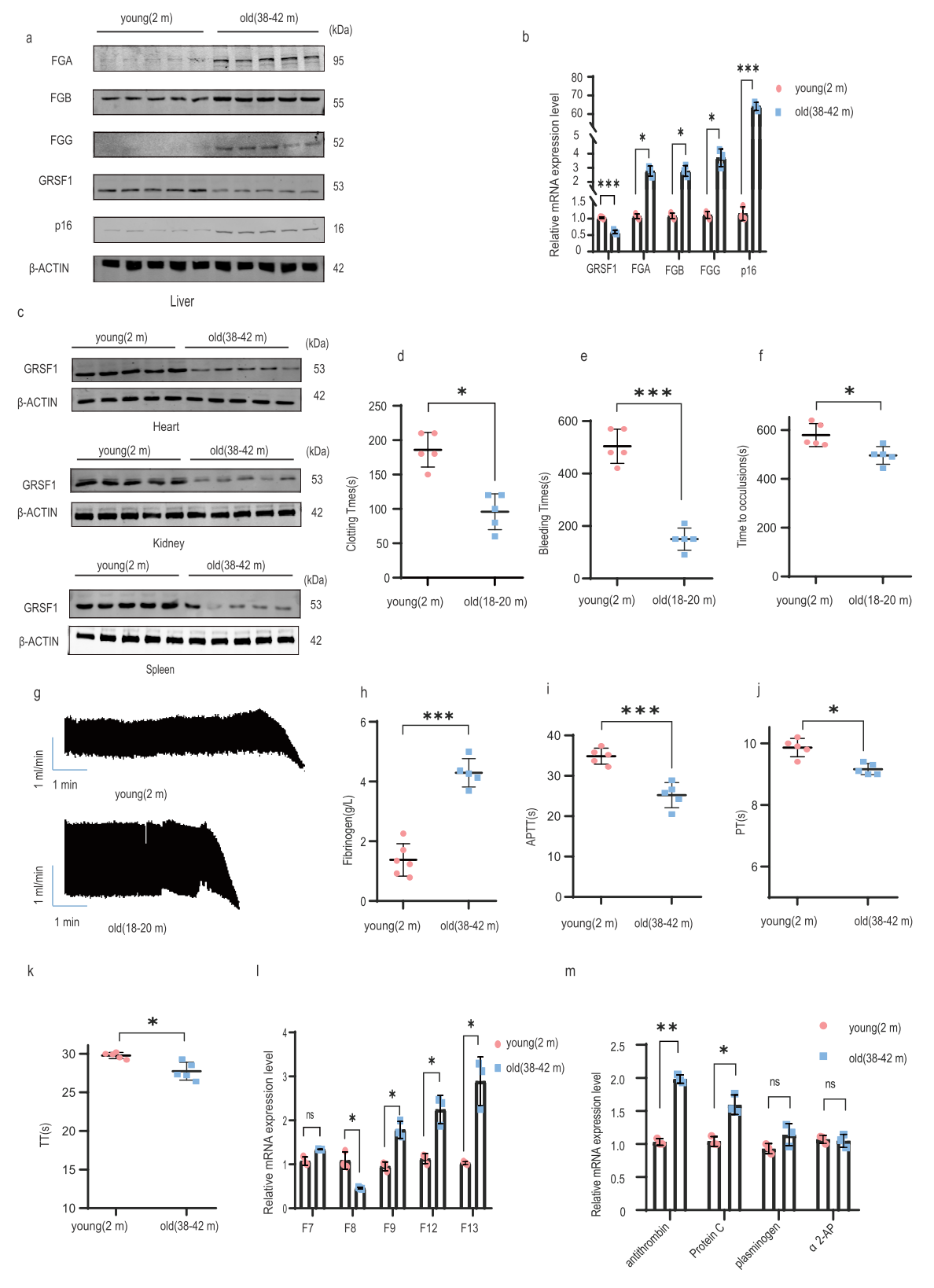


**Fig. S6 GRSF1 expression level decreases in multiple tissues, while many liver-produced coagulation factors mRNA levels increase, during BALB/c female mice aging. (related to Fig. 5)**

(A and B) GRSF1 expression level decreases in old mice liver. The total tissue lysates and RNAs of liver from young and old BALB/c female mice were extracted and subjected to WB and RT-qPCR analysis. (C) GRSF1 expression levels decrease in multiple tissues in old mice. The total tissue lysates of heart, kidney, spleen from young and old BALB/c female mice were extracted and subjected to WB analysis. (D) Blood clotting time in young and old mice. (E) Tail bleeding time in young and old mice. (F and G) Representative Doppler echocardiogram of the blood flow rate in mouse right carotid arteries following FeCl_3_ injury (F). The time when the flow rate was reduced to 0 mL/min was considered as the complete occlusion time. Statistic results of the right carotid artery complete occlusion time (G). (H-K) Serum fibrinogen concentration (H), APTT, PT, and TT of the platelet-free plasma isolated from young and old BALB/c female mice were measured by using corresponding kits. (L and M) The total RNAs of liver from young and old BALB/c female mice were extracted and subjected to RT-qPCR analysis for multiple liver-produced coagulation factors, anticoagulant proteins, and fibrinolytic system proteins.


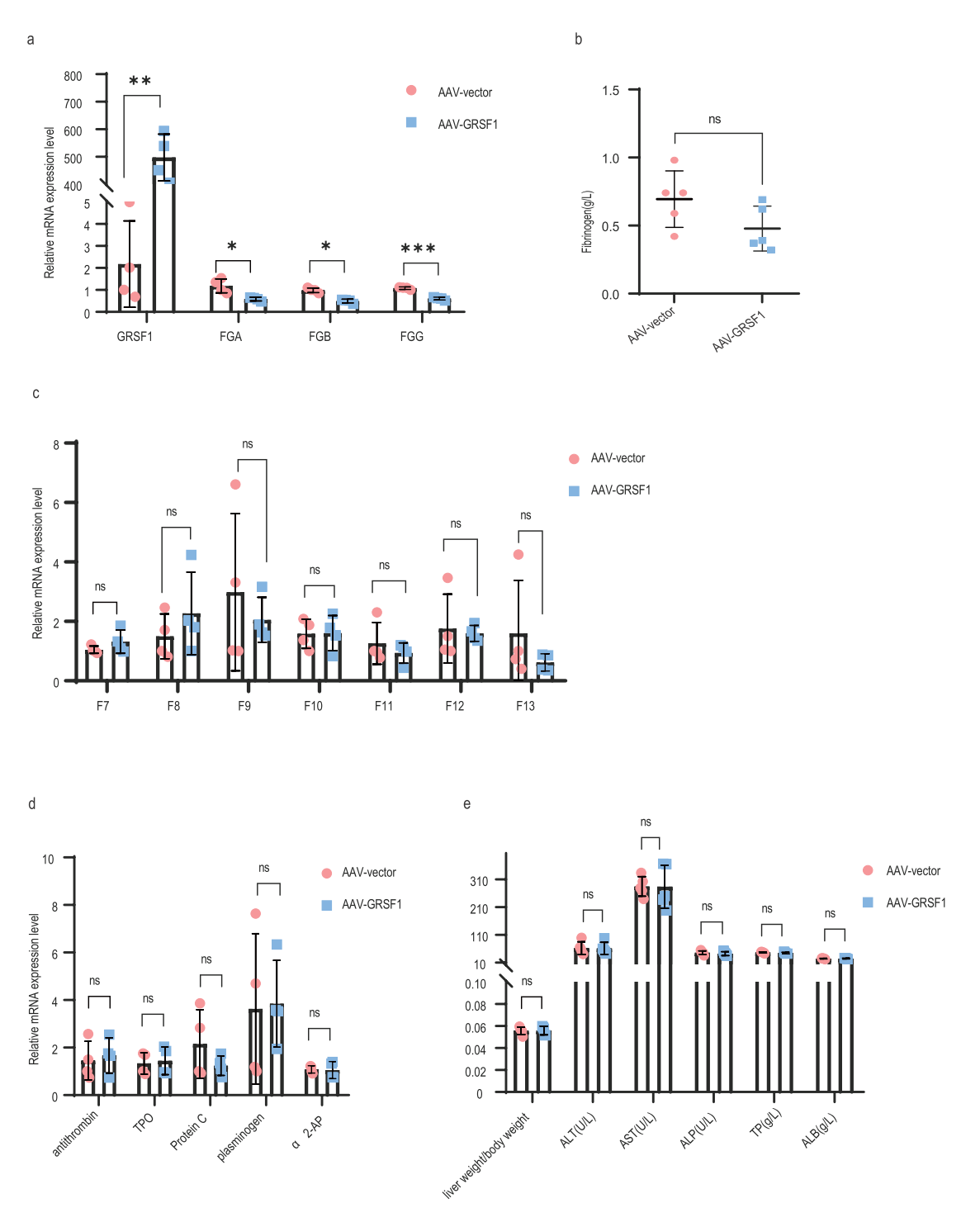


**Fig. S7 GRSF1 overexpression decreases fibrinogen mRNA and plasma levels without affecting other blood factors and causing liver damage in old mice. (related to Fig. 6)**

(A) Old BALB/c female mice (19-26 months) were infected by the indicated AAV9 virus, respectively. After 9 weeks AAV9 infection, the GRSF1 and fibrinogen mRNAs levels in mice liver were analyzed by RT-qPCR. (B) Fibrinogen plasma concentration was detected by ELISA kit. (C and D) The total RNAs of liver from old BALB/c female mice were extracted and subjected to RT-qPCR analysis for multiple liver-produced coagulation factors, anticoagulant proteins, and fibrinolytic system proteins. (E) Liver weight/body weight ratio, liver function parameters including ALT, AST, ALP, TP, and ALB in blood plasma were determined.


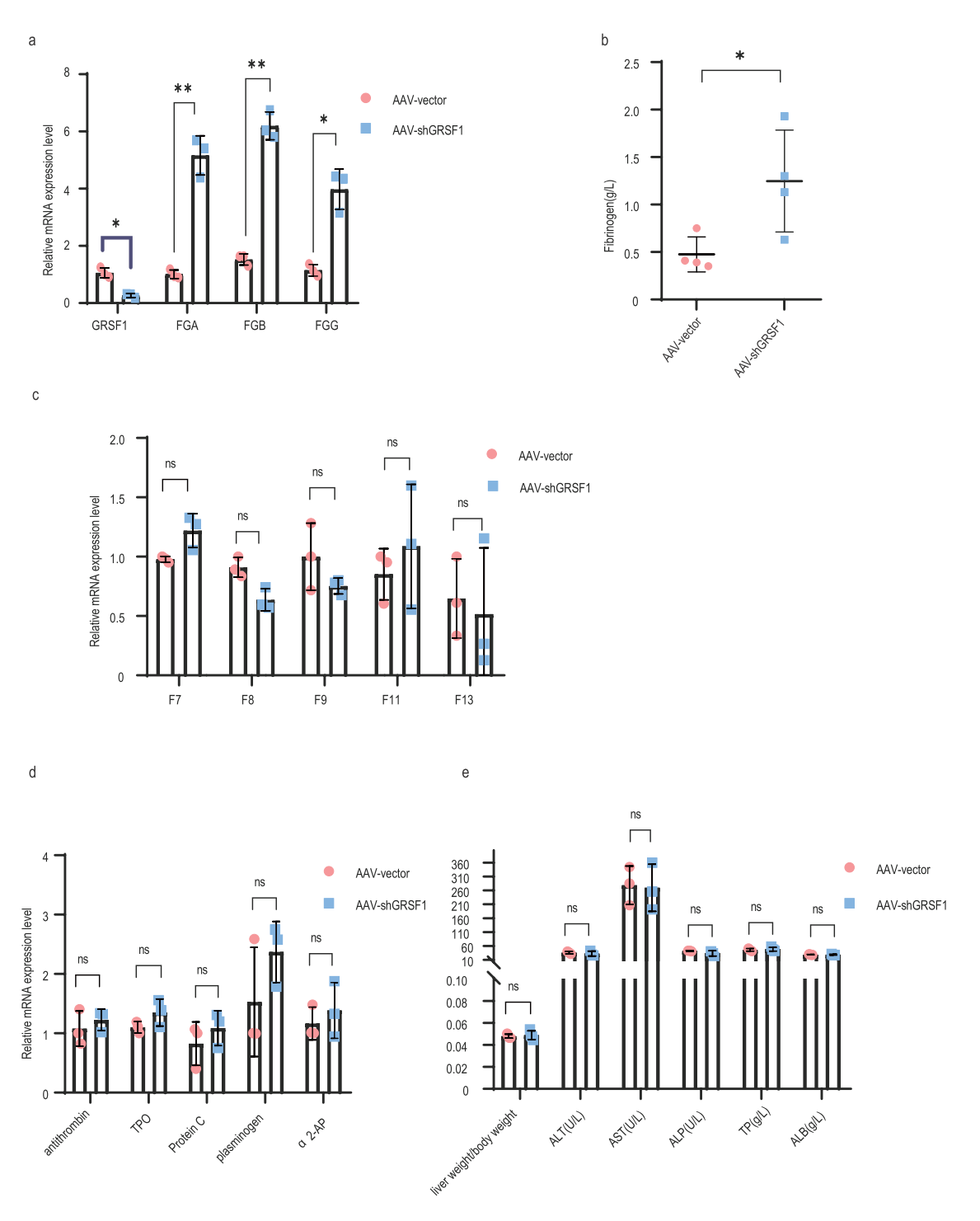


**Fig. S8 GRSF1 knockdown increases fibrinogen mRNA and plasma levels without affecting other blood factors and causing liver damage in young mice. (related to Fig. 7)**

(A) 2 months young BALB/c mice were infected by the indicated AAV9 virus, respectively. After 9 weeks AAV9 infection, the GRSF1 and fibrinogen mRNAs levels in mice liver were analyzed by RT-qPCR. (B) Fibrinogen plasma concentration was detected by ELISA kit. (C and D) The total RNAs of liver from young BALB/c mice were extracted and subjected to RT-qPCR analysis for multiple liver-produced coagulation factors, anticoagulant proteins, and fibrinolytic system proteins. (E) Liver weight/body weight ratio, liver function parameters including ALT, AST, ALP, TP, and ALB in blood plasma were determined.
