# Supplementary material for: GRSF1 antagonizes age-associated hypercoagulability via modulation of fibrinogen mRNA stability
Source: Cell Death Dis. 2023 Nov 3;14(11):717. doi: 10.1038/s41419-023-06242-9 (PMC10624831; doi:10.1038/s41419-023-06242-9)
Supplement: Supplementary file 2 — Original Data File [file 41419_2023_6242_MOESM2_ESM.pdf]

Figure 1

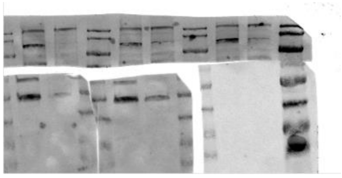

FGA, FGB, FGG

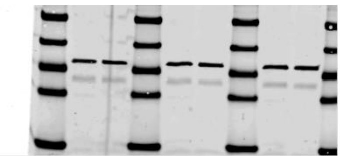

GAPDH

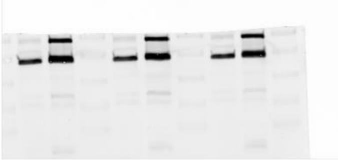

GRSF1

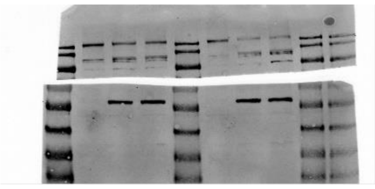

FGA, FGB

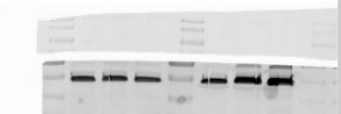

FGG

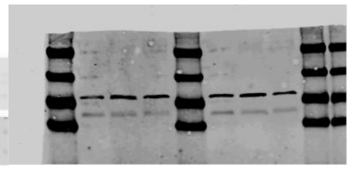

GAPDH

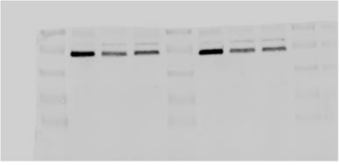

GRSF1

Figure 2

GRSF1

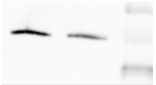

siGRSF1

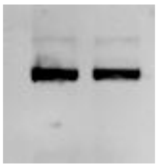

GAPDH

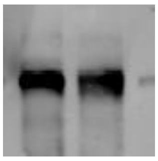

GAPDH

Figure 3

3C:

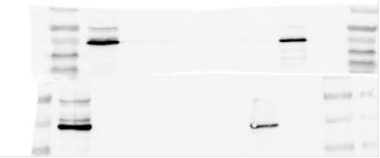

FGA, FGB

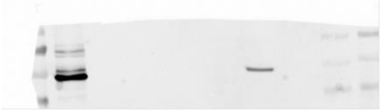

FGG

3d:

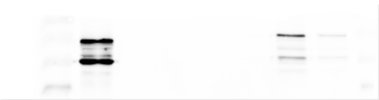

FGA

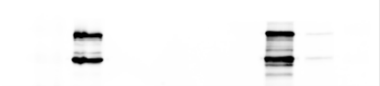

FGB

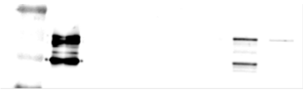

FGG

3e:

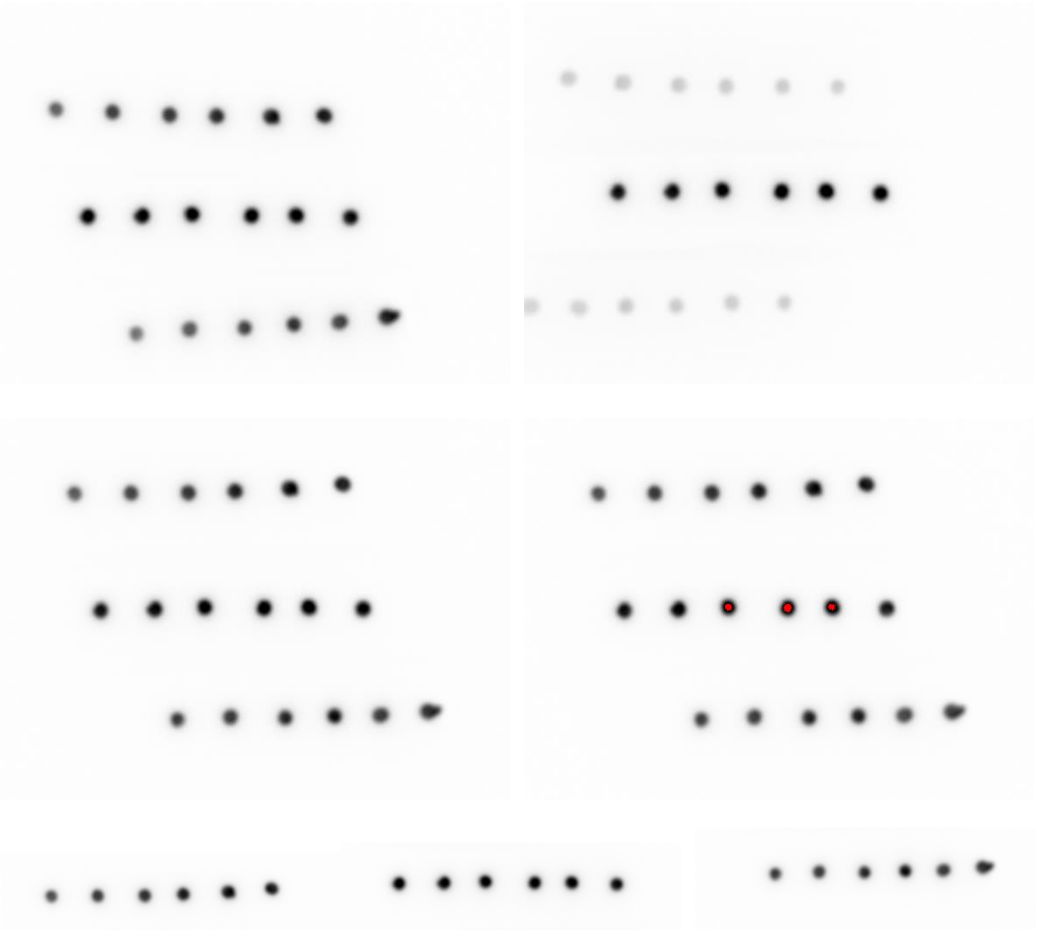

FGA FGB FGG

Figure 4

4b

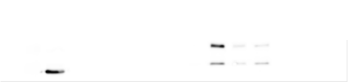

4c

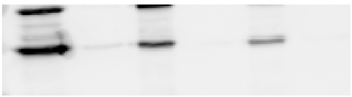

4d

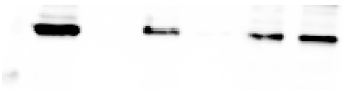

4e

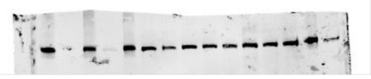

4g

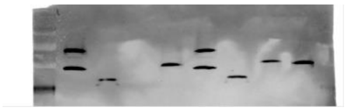

4h

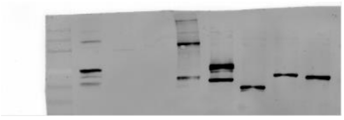

4i

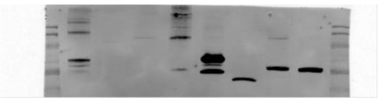

Figure 5

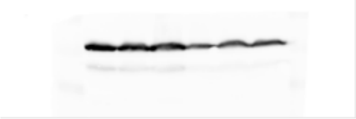

FGA

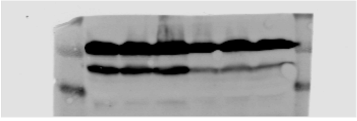

FGB

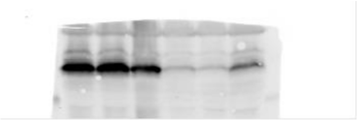

FGG

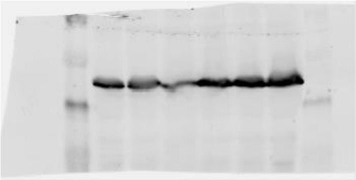

GRSF1

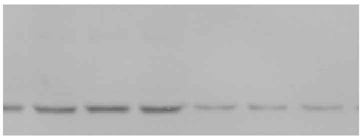

p16

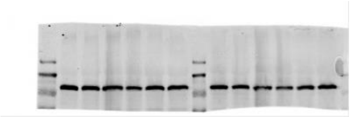

actin

Figure 6

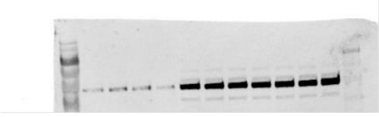

GRSF1

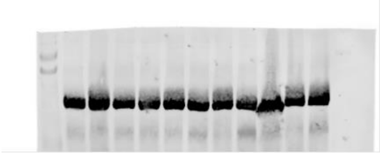

actin

Figure 7

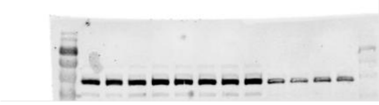

GRSF1

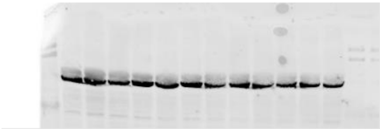

actin

Supplementary Figures
Fig S1

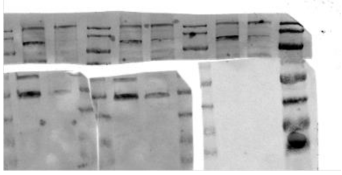

FGA, FGB, FGG

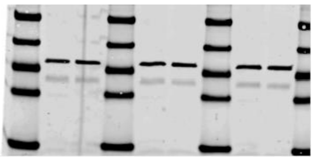

GAPDH

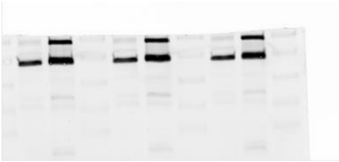

GRSF1

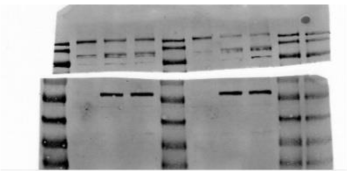

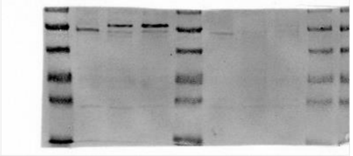

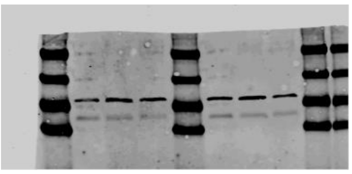

FGA, FGB

FGG

GAPDH

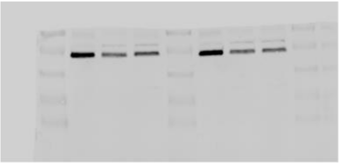

GRSF1

Fig S3

3b

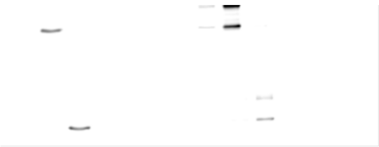

3c

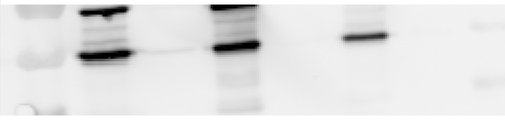

3d

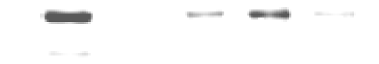

3e

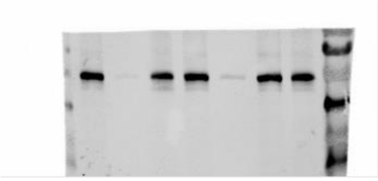

Fig S4

4b

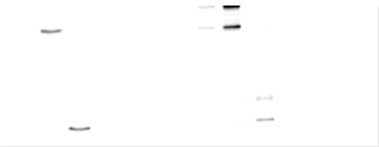

4c

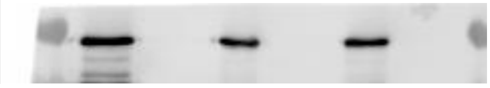

4d

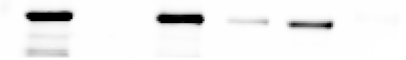

4e

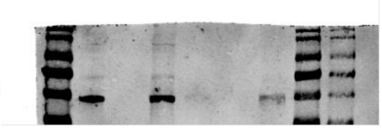

Fig S5

5c:

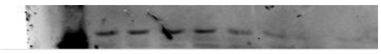

GRSF1

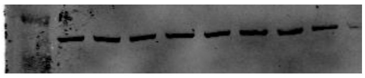

actin

5d:

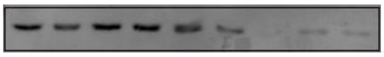

GRSF1

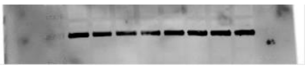

actin

5e:

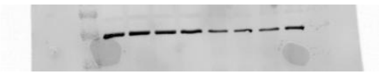

GRSF1

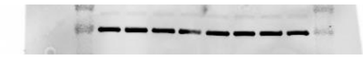

actin

Fig S6

6a:

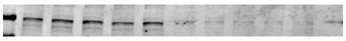

FGA

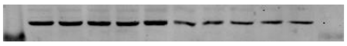

FGB

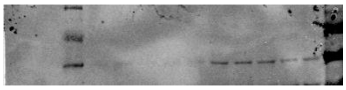

FGG

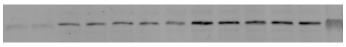

GRSF1

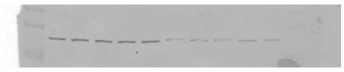

p16

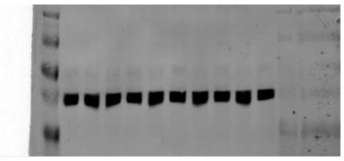

actin

6c:

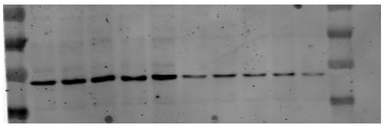

Heart-GRSF1

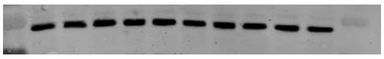

heart-actin

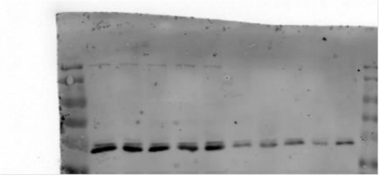

kidney-GRSF1

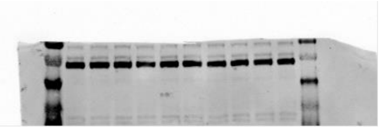

kidney-actin

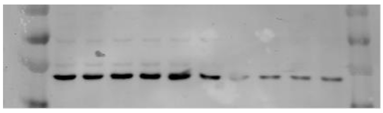

spleen-GRSF1

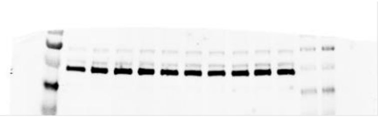

spleen-actin
